# LidSonic V2.0: A LiDAR and Deep-Learning-Based Green Assistive Edge Device to Enhance Mobility for the Visually Impaired

**DOI:** 10.3390/s22197435

**Published:** 2022-09-30

**Authors:** Sahar Busaeed, Iyad Katib, Aiiad Albeshri, Juan M. Corchado, Tan Yigitcanlar, Rashid Mehmood

**Affiliations:** 1 Faculty of Computer and Information Sciences, Imam Mohammad Ibn Saud Islamic University, Riyadh 11564, Saudi Arabia; 2 Department of Computer Science, Faculty of Computing and Information Technology, King Abdulaziz University, Jeddah 21589, Saudi Arabia; 3 Bisite Research Group, University of Salamanca, 37007 Salamanca, Spain; 4 Air Institute, IoT Digital Innovation Hub, 37188 Salamanca, Spain; 5 Department of Electronics, Information and Communication, Faculty of Engineering, Osaka Institute of Technology, Osaka 535-8585, Japan; 6 School of Architecture and Built Environment, Queensland University of Technology, 2 George Street, Brisbane, QLD 4000, Australia; 7 High Performance Computing Center, King Abdulaziz University, Jeddah 21589, Saudi Arabia

**Keywords:** visually impaired, smart mobility, sensors, LiDAR, ultrasonic, deep learning, obstacle detection, obstacle recognition, assistive tools, edge computing, green computing, sustainability, Arduino Uno, smart app

## Abstract

Over a billion people around the world are disabled, among whom 253 million are visually impaired or blind, and this number is greatly increasing due to ageing, chronic diseases, and poor environments and health. Despite many proposals, the current devices and systems lack maturity and do not completely fulfill user requirements and satisfaction. Increased research activity in this field is required in order to encourage the development, commercialization, and widespread acceptance of low-cost and affordable assistive technologies for visual impairment and other disabilities. This paper proposes a novel approach using a LiDAR with a servo motor and an ultrasonic sensor to collect data and predict objects using deep learning for environment perception and navigation. We adopted this approach using a pair of smart glasses, called LidSonic V2.0, to enable the identification of obstacles for the visually impaired. The LidSonic system consists of an Arduino Uno edge computing device integrated into the smart glasses and a smartphone app that transmits data via Bluetooth. Arduino gathers data, operates the sensors on the smart glasses, detects obstacles using simple data processing, and provides buzzer feedback to visually impaired users. The smartphone application collects data from Arduino, detects and classifies items in the spatial environment, and gives spoken feedback to the user on the detected objects. In comparison to image-processing-based glasses, LidSonic uses far less processing time and energy to classify obstacles using simple LiDAR data, according to several integer measurements. We comprehensively describe the proposed system’s hardware and software design, having constructed their prototype implementations and tested them in real-world environments. Using the open platforms, WEKA and TensorFlow, the entire LidSonic system is built with affordable off-the-shelf sensors and a microcontroller board costing less than USD 80. Essentially, we provide designs of an inexpensive, miniature green device that can be built into, or mounted on, any pair of glasses or even a wheelchair to help the visually impaired. Our approach enables faster inference and decision-making using relatively low energy with smaller data sizes, as well as faster communications for edge, fog, and cloud computing.

## 1. Introduction

There are over 1 billion disabled people today around the world, comprising 15% of the world population, and this number is greatly increasing due to ageing, chronic diseases, and poor environments and health according to the World Health Organization (WHO) [1]. WHO defines disability as having three dimensions, “impairment in a person’s body structure or function, or mental functioning; activity limitation; and participation restrictions in normal daily activities”, and states that disability “results from the interaction between individuals with a health condition with personal and environmental factors” [2]. Cambridge Dictionary defines disability as “not having one or more of the physical or mental abilities that most people have” [3]. Wikipedia defines physical disability as “a limitation on a person’s physical functioning, mobility, dexterity, or stamina” [4]. Disabilities can relate to various human functions, including hearing, mobility, communication, intellectual ability, learning, and vision [5]. In the UK, 14.6 million people are disabled [6], forming over 20% of the population. In the US, 13.2% of the population were disabled according to the 2019 statistics, comprising over 43 million people [7]. Similar statistics could be found in various countries around the world, some worse than others, which means that, on average, disabled people make up 15% of the population globally.

With 253 million people affected by visual impairment and blindness around the globe, it is the second most prevalent disability in the world population after hearing loss and deafness [8]. Four terminologies can be used to identify various rates of loss of vision and blindness, namely, partially sighted, low vision, legally blind, and totally blind [9]. People with partial vision in one or both eyes are considered partially sighted. Low vision relates to a serious visual impairment, where visual acuity in the good-seeing eye is 20/70 or lower and cannot be enhanced with glasses or contact lenses. If the best-seeing eye can be corrected to achieve 20/200, then the person is considered legally blind [9]. Finally, people who are totally blind are those with a total loss of vision [10]. Even though vision impairment can happen at any point in life, it is more common among older people. Visual impairment can be hereditary. In these kinds of circumstances, it occurs from birth or in childhood [11].

While visual impairment and blindness are among the most disabling disabilities, we know relatively little about the lives of visually impaired and blind individuals [12]. The WHO predicts that the number of people with visual impairments will increase owing to population growth and aging. Moreover, contemporary lifestyles have spawned a multitude of chronic disorders that degrade vision and other human functions [13]. Diabetes and hyperglycemia, for instance, can cause a range of health issues, including visual impairment. Several tissues of the ocular system can be affected by diabetes, and cataracts are one of the most prevalent causes of vision impairment [14].

Behavioral and neurological investigations relevant to human navigation have demonstrated that the way in which we perceive visual information is a critical part of our spatial representation [15]. It is typically hard for visually impaired individuals to orient themselves and move in an unknown location without help [16]. For example, landplane tracking is a natural mobility task for humans, but it is an issue for individuals with poor or no eyesight. This capacity is necessary for individuals to avoid the risk of falling and to alter their position, posture, and balance [16]. Moving up and down staircases, low and high static movable obstacles, damp flooring, potholes, a lack of information about recognized landmarks, obstacle detection, object identification, and dangers are among the major challenges that visually impaired people confront indoors and outdoors [17,18].

The disability and visual impairment statistics of Saudi Arabia are also alarming. Around 3% of people in Saudi Arabia reported the presence of a disability in 2016 [19]. According to the General Authority for Statistics in Saudi Arabia, 46% of all the disabled in Saudi Arabia who have one disability are visually impaired or blind, and 2.9% of the Saudi population have disabilities amounting to extreme difficulty [20]. The information provided above highlights the urgent need for research in the development of assistive technologies for general disabilities, including visual impairment.

A white cane is the most popular tool used by visually impaired individuals to navigate their environments; nevertheless, it has a number of drawbacks. It requires physical contact with the environment [12], cannot detect barriers above the ground, such as ladders, scaffolding, tree branches, and open windows [21], and generates neuromusculoskeletal overuse injuries and syndromes that may require rehabilitation [22]. Moreover, the user of a white cane is sometimes ostracized for social reasons [12]. In the absence of appropriate assistive technologies, visually impaired individuals must rely on family members or other people [23]. However, human guides can be dissatisfying at times, since they may be unavailable when assistance is required [24]. The use of assistive technologies can help visually impaired and blind people engage with sighted people and enrich their lives [12].

Smart societies and environments are driving extraordinary technical advancements with the promise of a high quality of life [25,26,27,28]. Smart wearable technologies are generating numerous new opportunities in order to enhance the quality of life of all people. Fitness trackers, heart rate monitors, smart glasses, smartwatches, and electronic travel aids are a few examples. The same holds true for visually impaired people. Multiple devices have been developed and marketed to aid visually impaired individuals in navigating their environments [29]. An electronic travel aid (ETA) is a regularly used type of mobility-enhancing assistive equipment for the visually impaired and blind. It is anticipated that ETAs will increasingly facilitate “independent, efficient, effective, and safe movement in new environments” [30]. ETAs can provide information about the environment through the integration of multiple electronic sensors and have shown their effectiveness in improving the daily lives of visually impaired people [31]. ETAs are available in a variety of wearable and handheld devices and may be categorized according to their usage of cellphones, sensors, or computer vision [32]. The acceptance rate of ETAs is poor among the visually impaired and blind population [23]. Their use is not common among potential users because they have inadequate user interface designs, are restricted to navigation purposes, are functionally complex, weighty to carry, expensive, and lack functionality for object recognition, even in familiar indoor environments [23]. The low adoption rate does not necessarily indicate that disabled people oppose the use of ETAs; rather, it confirms that additional research is required to investigate the causes of the low adoption rate and to improve the functionality, usability, and adaptability of assistive technologies [33]. In addition, the introduction of unnecessarily complicated ETAs that may necessitate extensive and supplementary training to learn additional and difficult abilities is not a realistic alternative and is not a feasible solution [22]. Robots can assist the visually impaired in navigating from one location to another, but they are costly, along with their other challenges [34]. Augmented reality has been used as a solution for magnifying text and images through a finger wearable applied with a camera to project on a HoloLens [35], but this technology is not suitable for blind people.

We developed a comprehensive understanding of the state-of-the-art requirements of, and solutions for, visually impaired assistive technologies using a detailed literature review (see Section 2) and a survey [36] of this topic. Using this knowledge, we identified the design space for assistive technologies for the visually impaired and the research gaps. We found that the design considerations for assistive technologies for the visually impaired are complex and include reliability, usability, and functionality in indoor, outdoor, and dark environments; transparent object detection; hand-free operations; high-speed, real-time operations; low battery usage and energy consumption; low computation and memory requirements; low device weight; and cost effectiveness. Despite the fact that several devices and systems for the visually impaired have been proposed and developed in academic and commercial settings, the current devices and systems lack maturity and do not completely fulfill user requirements and satisfaction [18,37]. For instance, numerous camera-based and computer-based solutions have been produced. However, the computational cost and energy consumption of image processing algorithms pose a concern for low-power portable or wearable devices [38]. These solutions require large storage and computational resources, including large RAMs to process large volumes of data containing images. This would require substantial processing, communication, and decision-making times, and would also consume energy and battery life. Significantly more research effort is required to bring innovation, intelligence, and user satisfaction to this crucial area.

In this paper, we propose a novel approach that uses a combination of a LiDAR with a servo motor and an ultrasonic sensor to collect data and predict objects using machine and deep learning for environment perception and navigation. We implemented this approach using a pair of smart glasses, called LidSonic V2.0, to identify obstacles for the visually impaired. The LidSonic system consists of an Arduino Uno edge computing device integrated into the smart glasses and a smartphone app that transmits data via Bluetooth. Arduino gathers data, operates the sensors on smart glasses, detects obstacles using simple data processing, and provides buzzer feedback to visually impaired users. The smartphone application collects data from Arduino, detects and classifies items in the spatial environment, and gives spoken feedback to the user on the detected objects. LidSonic uses far less processing time and energy than image-processing-based glasses by classifying obstacles using simple LiDAR data and using several integer measurements.

We comprehensively describe the proposed system’s hardware and software design, having constructed their prototype implementations and tested them in real-world environments. Using the open platforms WEKA and TensorFlow, the entire LidSonic system was built with affordable off-the-shelf sensors and a microcontroller board costing less than USD 80. Essentially, we provide the design of inexpensive, miniature green devices that can be built into, or mounted on, any pair of glasses or even a wheelchair so as to help the visually impaired. Our approach affords faster inference and decision-making using relatively low energy with smaller data sizes. Smaller data sizes are also beneficial in communications, such as those between the sensor and processing device, or in the case of fog and cloud computing, because they require less bandwidth and energy and can be transferred in relatively shorter periods of time. Moreover, our approach does not require a white cane (although it can be adapted to be used with a white cane) and, therefore, it allows for handsfree operation.

The work presented in this paper is a substantial extension of our earlier system LidSonic (V1.0) [39]. LidSonic V2.0, the new version of the system, uses both machine learning and deep learning methods for classification, as opposed to V1.0, which uses machine learning alone. LidSonic V2.0 provides a higher accuracy of 96% compared to 92% for LidSonic V1.0, despite the fact that it uses a lower number of data features (14 compared to 45) and a wider vision angle of 60 degrees compared to 45 degrees for LidSonic V1.0. The benefits of a lower number of features are evident in LidSonic V2.0, requiring even lower computing resources and energy than LidSonic V1.0. We have extended the LidSonic system with additional obstacle classes, provided an improved and extended explanation of its various system components, and conducted extensive testing with two new datasets, six machine learning models, and two deep learning models. System V2.0 was implemented using the Weka and TensorFlow platforms (providing dual options for open-source development) compared to the previous system that was implemented using Weka alone. Moreover, this paper provides a much extended, completely new literature review and taxonomy of assistive technologies and solutions for the blind and visually impaired.

Earlier in this section, we noted that, despite the fact that several devices and systems for the visually impaired have been developed in academic and commercial settings, the current devices and systems lack maturity and do not completely fulfill user requirements and satisfaction. Increased research activity in this field will encourage the development, commercialization, and widespread acceptance of devices for the visually impaired. The technologies developed in this paper are of high potential and are expected to open new directions for the design of smart glasses and other solutions for the visually impaired using open software tools and off-the-shelf hardware.

The paper is structured as follows. Section 2 explores relevant works in the field of assistive technologies for the visually impaired and provides a taxonomy. Section 3 gives an overview of the LidSonic V2.0 system, highlighting its user, developer, and system features. Section 4 provides a detailed illustration of the software and hardware design and implementation. The system is evaluated in Section 5 Conclusions and thoughts regarding future work are provided in Section 6.

## 2. Related Work

**Figure 1:**
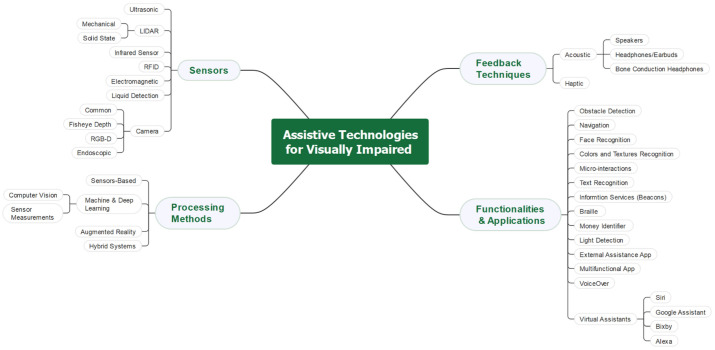
A Taxonomy of Research on Assistive Technologies for the Visually Impaired.

This section reviews the literature relating to this paper. Section 2.1 presents the sensor technologies and the types of sensor technologies used in the assistive tools for the visually impaired. Section 2.2 reviews the processing methods. Section 2.3 discusses the feedback techniques. A taxonomy of functions and applications is provided in Section 2.4. Section 2.5 identifies the research gap and justifies the need for this research. A taxonomy of the research on the visually impaired presented in this section is given in Figure 1. An extensive complimentary review of the assistive technologies for the visually impaired and blind can be found in our earlier work [39].

### 2.1. Sensor Technologies and Types Used in Assistive Tools

Sensors are indistinguishable components of cyberphysical systems. They collect knowledge regarding environmental factors, as well as non-electrical system parameters, and provide the findings as electrical signals. With the development of microelectronics, sensors are available as compact devices at low costs and have a wide range of applications in different fields, especially in control systems [40]. There are two types of sensors, including passive and active sensors. The active sensor needs an incentive to activate. On the contrary, the passive sensor detects inputs and generates output signals directly without an external incentive. The classification may be dependent on the sensor’s means of detection, e.g., electric, biological, chemical, radioactive, etc. [41].

A number of sensors have been employed in the field of technologies for visually impaired people. They have been used to solve a wide range of vision issues. The most frequent types of sensors used in assistive devices for the visually impaired are listed in Table 1. It also shows the sensors’ functions (functionality), types of wearables, and types of feedback.

#### 2.1.1. Ultrasonic Sensors

An ultrasonic sensor is an electronic device that uses ultrasonic sound waves to detect the distance between the user and a target item and transforms the reflected sound into an electric signal. Fauzul and Salleh [42] developed a visual assistive technology to assist visually impaired individuals in safely and conveniently navigating both indoor and outdoor situations. A smartphone app and an obstacle sensor are the two major components of the system. To deliver auditory cue instructions to the user, the mobile software makes use of the smartphone’s microelectromechanical sensors, location services, and Google Maps. Ultrasonic sensors in the obstacle sensor are used to detect objects and offer tactile feedback. The obstacle avoidance gadget attaches to a standard cane and vibrates the handle with varying intensities according to the nature of the obstructions. The spatial distance and direction from the present position to the intended location are used to produce spatial sound cues. Gearhart et al. [43] proposed a technique for identifying the position of the detected object using triangulation by geometric relationships with scalar measurements. The authors placed two ultrasonic sensors one on each shoulder, which were angled towards each other at five degrees from parallel, with a space of 10 inches. However, this technique is too complex to be applied to several objects in front of the sensor. A significant number of research papers in the field of objects detection have depended on ultrasonic sensors. Tudor et al. [44] proposed a wearable belt with two ultrasonic sensors and two vibration motors to direct the visually impaired away from obstacles. The authors used an Arduino Nano Board with an ATmega328P microcontroller to connect and build their system. According to their findings, the authors in [45] found that ultrasonic sensors and vibrator devices are easily operated by Arduino UNO R3Impatto Zero boards. Noman et al. The authors of [46] proposed a robot equipped with several ultrasonic sensors and Arduino Mega (ATMega 2560 processor) to detect obstacles, holes, and stairs. The robots can be utilized in indoor environments; however, their use outdoors is not practical.

#### 2.1.2. LiDAR Sensors

Light detection and ranging, or LiDAR, is a common remote sensing technique used for determining an object’s distance. Chitra et al. [47] proposed a handsfree LVU (LiDARs and Vibrotactile Units) discrete wearable gadget that helps blind persons to identify impediments. Proper mobile assistance equipment is required. The proposed gadget consists of a wearable sensor strap. Liu et al. proposed HIDA. This is a lightweight assistance system used for comprehensive indoor detection and avoidance based on 3D point cloud instance segmentation and a solid-state LiDAR sensor. The authors created a point cloud segmentation model with dual lightweight decoders for semantic and offset predictions, ensuring the system’s efficiency. The segmented point cloud was post-processed by eliminating outliers and projecting all points onto a top-view 2D map representation after the 3D instance segmentation.

#### 2.1.3. Infrared (IR) Sensors

An infrared (IR) sensor is an electronic device that monitors and senses the infrared radiation in its surroundings [48]. Infrared signals are similar to RFID in that they rely on distant data transfer. As previously stated, the latter uses radio waves, whilst the former uses light signals. Air conditioner remotes and motion detectors, for example, all use infrared technology. Smartphones now come with infrared blasters, allowing users to control any compatible device with an infrared receiver. It is an eye-safe light, which emits pulses and measures the time taken to calculate the distance using the reflected light. Every metric of the IR consists of thousands of separate pulses of light that lead to reliable measurements of rain, snow, fog, or dust and can be obtained by an infrared sensor. These measurements are difficult to capture with cameras [22]. In addition, IR has a long range in both indoor and outdoor environments, high precision, small size, and low latency. An IR sensor can detect obstacles up to 14 m away, with a 0.5 resolution and 4 cm accuracy [12]. IR has medium width among the ultrasonic and laser sensors. The laser has a rather narrow scope, and it gathers a very limited amount of space information, which is not large enough for free paths. On the other hand, ultrasonic sensors have many reflections; thus, they are limited [49].

#### 2.1.4. RFID Sensors

RFID is the abbreviation of radio frequency identification. Data may be transferred and received through radio waves using RFID. In RFID, the sender sends a radio receiver. In this method, the sender is commonly an RFID chip (or an RFID tag) inserted into the object being read or scanned. The receiver, on the other hand, is an electrical device that detects the RFID chip’s data. The chip and receiver do not need to be in physical contact, because the data is broadcast and received by radio waves. RFID is appealing because of its remote capabilities, but it is also harmful. Because the chip’s RFID signal may be read by anybody with an RFID reader, it may lead to an unethical and harmful situation, namely, data theft. The fact that the person using the scanner does not even have to be near the chip/tag increases the danger. Another disadvantage is that each tag has a certain range, which necessitates extensive individual testing, limiting the scope. In addition, the system may be quickly turned off if the tags are wrapped or covered, preventing them from receiving radio signals [50].

An intelligent walking stick for the blind was proposed by Chaitrali et al. [51]. The proposed navigation system for vision impairment uses infrared sensors, RFID technology, and Android handsets to provide speech output for the purpose of obstacle navigation. The gadget is equipped with proximity infrared sensors, and RFID tags are implanted in public buildings, as well as in the walking sticks of blind people. The gadget is Bluetooth-connected to an Android phone. An Android application that provides voice navigation based on the RFID tag reading and also updates the server with the user’s position information is being developed. Another application allows family members to access the location of the blind person via the server at any time. The whereabouts of a blind person may be traced at any time, providing further security. This approach has the disadvantage of not being compact. When the intelligent stick is within the range of the PCB unit, the active RFID tags immediately send location information. It is not necessary for the RFID sensor to read it explicitly.

#### 2.1.5. Electromagnetic Sensors (Microwave Radar)

Adopting a pulsed chirp scheme can reduce the power consumption and preserve a high resolution by managing the spatial resolution in terms of the frequency modulation bandwidth. A pulsed signal enables the transmitter to be turned off in the listening time of the echo, thereby significantly reducing the energy consumption [37]. Using a millimeter-wave radar and a typical white cane, a method of electronic travel assistance for blind and visually impaired persons was proposed [52]. It is a sophisticated system that not only warns the user of possible difficulties but also distinguishes between human and nonhuman targets. Because real-world situations are likely to include moving targets, a novel range alignment approach was developed to detect minute chest movements caused by physiological activity as vital evidence of human presence. The proposed system recognizes humans in complicated situations, with many moving targets, giving the user a comprehensive set of information, including the presence, location, and type of the accessible targets. The authors used a 122 GHz radar board to carry out appropriate measurements, which were used to demonstrate the system’s working principle and efficacy.

#### 2.1.6. Liquid Detection Sensors

Research usually involves more than one type of sensor in order to cover most of the prevalent challenges facing the visually impaired. Ikbal et al. [53] proposed a stick that is equipped with various kinds of sensors to assist in detecting obstacles. One of the major obstacles that jeopardize the visually impaired is water on the floor [54]. Therefore, the authors included a water sensor in their solution, in addition to two ultrasonic sensors to detect 180 cm obstacles, including one IR sensor to detect stairway gaps and holes on streets, and a temperature sensor for fire alert. The authors connected all the sensors with an Arduino microcontroller board. This sensor must come into contact with the surface of the water in order to provide the result; thus, this method must consider the appropriate wearable. One of the most important uses of a liquid detector for the blind is the use of a sensor that is placed on the cup to prevent it from spilling. To improve navigation safety, the authors present a polarized RGB-Depth (pRGB-D) framework to detect the traversable area and water hazards while using polarization-color-depth-attitude information [55].

#### 2.1.7. Cameras

The camera is utilized to provide various functions in different technology solutions using machine learning algorithms, such as facial recognition, object recognition, and localization (see Table 1). Research has used various types of cameras. The most frequently used types are the common camera and the RGB-Depth camera. The common camera is used mostly in facial, emotion, and obstacle recognition. On the other hand, the RGB-D camera has been used for detecting and avoiding obstacles and mapping to assist in navigation through indoor environments. A depth image is an image channel in which each pixel is related to a distance between the image plane and the respective point in the RGB picture. Adding depth to standard color camera techniques increases both the precision and density of the map. RGB-D sensors are popular in a variety of visual aid applications due to their low power consumption and inexpensive cost, as well as their resilience and high performance, as they can concurrently sense color and depth information at a smooth video framerate. Because polarization characteristics reflect the physical properties of materials, polarization and associated imaging can be employed for material identification and target detection, in addition to color and depth [30]. Meanwhile, because various polarization states of light act differentially at the interface of an object’s surface, polarization has been utilized in a variety of surface measuring techniques. Nevertheless, most industrial RGB-D sensors, such as light-coding sensors and stereo cameras, depend solely on intensity data, with polarization indications either missing or insufficient [55]. The study reported in [56] describes a 3D object identification algorithm and its implementation in a robotic navigation aid (RNA) that allows for the real-time detection of indoor items for blind people utilizing a 3D time-of-flight camera for navigation. Then, using a Gaussian-mixture-model-based plane classifier, each planar patch is classified as belonging to a certain object model. Finally, the categorized planes are clustered into model objects using a recursive plane clustering process. The approach can also identify various non-structural elements in the indoor environment. The authors of the research reported in [57] proposed a new approach to autonomous obstacle identification and classification that combines a new form of sensor, a patterned light field, with a camera. The proposed gadget is compact in terms of size, portable, and inexpensive. As the sensor system is transported in natural interior and outdoor situations over and toward various sorts of barriers, the grid projected by the patterned light source is visible and distinguishable. The proposed solution uses deep learning techniques, including a convolutional neural-network-based categorization of individual frames, to leverage these patterns without calibration. The authors improved their method by smoothing frame-based classifications across many frames using lengthy short-term memory units.

**Table 1:** Types of Sensors Used in Assistive Devices for VI.

Sensor Name	Works	Purpose of Sensor	No. of Sensors	Weight	Wearable/Assistive	Feedback Method
IR Sensors	[58]	Touch down, touch up sensor	2	Light	Mounted on top of a finger	Acoustic
	[49]	Detect obstacles, stairs	2	Light	Cane	Acoustic
IMU	[58]	Recognize gestures and sense movements	1	Light	Mounted on top of a finger	Acoustic
Ultrasonic Sensors	[23]	Detect obstacles up to the chest level	5	Light	Cane	Acoustic, vibration
	[59]	Detect obstacles	2	High	Guide dog robot and portable robot	Acoustic
	[45]	Detect obstacles	5	Fair	Mounted on the head, legs, and arms	Buzzer, vibration
	[44]	Detect obstacles	2	Fair	Belt	Vibration
ToF Distance Sensors	[12]	Detect obstacles	7	High	Belt	Vibration belt
Microwave Radar	[37]	Detect obstacles	1	Light	Mounted on a cane	Acoustic, vibration
Wet Floor Detection Sensors	[53]	Detect wet floors	1	Light	Cane	Buzzer
Bluetooth	[60]	Informing about indoor environments	3	Light	Beacon transmitter, smartphone	Acoustic
Laser Pointer	[61]	Detect obstacles	1	Light	Belt	Vibration belt
Cameras	[58]	Localize the hand touch	1	Light	Mounted on top of a finger	Acoustic
	[62]	Emotion recognition	1	Light	Clipped on to spectacles	Vibration belt
	[59]	Obstacle recognition (traffic light, cones, bus, etc.)	2	High	Guide dog robot and portable robot	Acoustic
	[63]	Localization system	1	Low	Head level (helmet), chest level (hanged)	Location in a Map
RGB-D Cameras	[61]	Detect obstacles	1	Light	Smartphone simulating a cane	Vibratory belt
	[64]	Avoid obstacles, localization system for indoor navigation	1	Light	Glass, tactile vest, smartphone	Haptic vest (4 vibration motors)
Endoscopic Cameras	[65]	Identify clothing colors, visual texture recognition	1	Light	Mounted on top of a finger	Acoustic
Compass	[66]	Indoor navigation	1	Light	Optical head-mounted (Glass)	Acoustic

### 2.2. Processing Methods

Researchers have used a range of processing methods for assistive technologies. Recent years have seen an increase in the use of machine learning and deep learning methods in various applications and sectors, including healthcare [67,68,69], mobility [70,71,72], disaster management [73,74], education [75,76], governance [77], and many other fields [78]. Assistive technologies are no different and have begun to increasingly rely on machine learning methods. This section reviews some of the works on processing methods for assistive technologies, including both machine learning-based methods and other methods.

There are numerous ideas and methods that have been proposed to solve the problems and challenges facing the blind. Katzschmann et al. [12] incorporated several sensors and feedback motors in a belt to produce an aiding navigation system, called Array of LiDARs and Vibrotactile Units (ALVU), for visually impaired people. The authors developed a secure navigation system, which is effective in providing detailed feedback to a user about the obstacles and free areas around the user. Their technology is made up of two components: a belt with a distance sensor array and a haptic array of feedback modules. The haptic strap that goes around the upper abdomen provides input to the person wearing the ALVU, which allows them to sense the distance between themselves and their surroundings. As the user approaches an impediment, they receive greater pulse rates and a higher vibration force. The vibration and pulses stop once the user has overcome the obstacle. However, this kind of feedback is primitive and cannot define the type of obstacle that the user should avoid. Moreover, it does not determine whether the obstacle should or should not be avoided. In addition, wearing two belts may not be easy and comfortable for the user. Meshram et al. [23] designed a NavCane that detects and avoids obstacles from the floor up to the chest level. It can also identify water on the floor. It has a user button to send auto alerts through SMS and email in emergencies. It provides two kinds of feedback, including tactile feedback using vibration and auditory feedback using the headphones. However, the device cannot identify the nature of the objects and cannot detect obstacles above chest level.

Hong et al. [79] proposed a solution for blind people based on two haptic wristbands used to provide feedback on objects. Using a LiDAR, Chun et al. [80] proposed a detection technique that reads the distances of deferent angles and then measures the predicted obstacles by comparing these reading.

Using the Internet of Things (IoT), machine learning, and embedded technologies, Mallikarjuna et al. [34] developed a low-cost visual aid system for object recognition. The image is acquired by the camera and then forwarded to the Raspberry Pi. To classify the image, the Raspberry Pi is trained using the TensorFlow Machine Learning Framework and Python programming. However, their technique requires a long period of time (5 s to 8 s) to inform the visually impaired individual about the item in front of them.

Gurumoorthy et al. [81] proposed a technique using the rear camera of a mobile phone to capture and analyze the image in front of the visually impaired. To execute tasks related to computer vision, this device uses Microsoft Cognitive Services. Then, image feedback is provided to the user through Google talkback. This technique needs a mobile internet service in order to be performed. Additionally, it is hard for the visually impaired to take a proper picture. A similar solution by means of sending the picture to the cloud to be analyzed was proposed in [33]; however, the authors captured the image through a camera mounted into the white cane. The authors also proposed a solution for improving visually impaired people’s mobility, which comprises a smart folding stick that works in tandem with a smartphone app using interconnection mechanisms based on GPS satellites. Navigational feedback is presented to the user as a voice output, as well as to the visually impaired family/guardians via the smartphone application. Rao and Singh [82] developed an obstacle detection method based on computer vision using a fisheye camera that is mounted onto a shoe. The photo is transmitted to a mobile application that uses TensorFlow Lite to classify the picture and alert visually impaired users about potholes, ditches, crowded places, and staircases. The device gives a vibration notification. In addition, an ultrasonic sensor is mounted with a servo on the front of the shoe to detect nearby obstacles. A vibration motor inside the shoe is used for feedback.

### 2.3. Feedback Techniques

People with standard vision depend on feedback that they gain from vision. They perceive more through vision than through hearing or touch. This is something that the visually impaired lack. Therefore, ETAs must be able to provide sufficient input on the perceived knowledge about the world of the user. Furthermore, feedback should be swift and not conflict with hearing and feeling [61].

#### 2.3.1. Haptics

The haptic methods for the visually impaired person can offer more methods for interaction with the other human senses, such as hearing, and do not interfere with them. It has been noted in studies that the visually impaired have higher memorization abilities and recognition of haptic tasks [83]. The advantages of haptic feedback are high privacy, because only the person can observe the stimuli, as well as usefulness in high-noise environments and the fact that they can expand the person’s experience as an additional communicational channel [84]. Buimer et al. [62] presented an experiment of a technique used to recognize facial emotion and send feedback through a vibration belt to the user. The authors conveyed information regarding six emotions by installing six vibration units in a belt. Even though the technique has accuracy problems, the satisfactory results of this method are based on a study of eight visually impaired people. Five of them found that the belt was easy to use and could interpret the feedback while conversing with another person. Meanwhile, the other three found its use difficult. Gonzalez-Canete et al. have proposed Tactons, whereby they identified sixteen applications with different vibration signals so that they could be distinguished from one another. The authors found that musical techniques for haptic icons are more recognizable and can be further distinguished. In addition, adding complicated vibrotactile sensations to smartphones is a significant benefit for users with any kind of sensory disability. The authors measured the recognition rate of the VI users and non-VI users. They found that non-VI users scored higher rates, especially with identification applications that they were familiar with, but when they used the reinforcement learning stage, in which some feedback is provided to the users, the recognition rate of the VI users increased [84].

#### 2.3.2. Acoustic

Masking auditory signals by binaurally re-displaying environmental information through headphones or earbuds blocks vital environmental signals on which many visually impaired people rely for secure navigation [22]. Currently, bone-transmitting helmets enable the user to obtain 3D auditory feedback, leaving the ear canal open and enabling the user to operate with free eyes, hands, and mind. The algorithm reduces sound production that does not indicate a change in order to further minimize the auditory output. Thus, the audible output sound is only produced when the user is confronted with an impediment, limiting possibly irritating sounds to a minimum [85].

### 2.4. Functions and Applications

Here, we review the necessary functions and applications that the visually impaired use to solve difficult matters. These applications are obstacle detection, navigation, facial recognition, color and texture recognition, micro-interactions, text recognition, informing services, and braille displayers and printers. Next, a detailed review is presented.

#### 2.4.1. Obstacle Detection

A great deal of research and many studies have focused on obstacle detection due to its significance for the visually impaired, as it is considered to be a major challenge for them. An ETA using a microwave radar to detect obstacles up to the head level through the vertical beam of the sensor was presented in [37]. To overcome the issue of power consumption, the authors switched off the transmitter during the listening time of the echo. Moreover, the pulsed chirp scheme was adapted to manage the spatial resolution. To improve the precision of the indoor blind guide robot’s obstacle recognition, Du et al. [86] presented a sensor data fusion approach based on the DS evidence theory of the genetic algorithm. The system uses ultrasonic sensors, infrared sensors, and LiDAR to collect data from the surroundings. The optimized weight is replaced in DS evidence theory by data fusion for the purpose of determining the weight range of various sensors using the genetic algorithm. In practice, weighing and fusing evidence requires the determination of the weight of the evidence. Their technique has an accuracy of 0.94 for indoor obstacle identification. Bleau et al. [87] presented EyeCane, which can identify four kinds of obstacles: cubes, doors, posts, and steps. However, its bottom sensor failed to properly identify objects on the ground, making downwards navigation more dangerous.

#### 2.4.2. Navigation

Navigation can be divided into two main categories, including internal and external navigation, because the set of techniques used in each one is different from the other. For example, the global positioning system (GPS) is not suitable for indoor localization due to the power of satellite signals, which become weak and cannot determine whether the user is close to a building or a wall [88]. However, some studies have developed techniques that may apply to both.

AL-Madani et al. [88] adopted a fingerprinting localization algorithm with fuzzy logic type-2 to navigate indoors in rooms with six BLE (Bluetooth low energy) beacons. The algorithm calculation was performed on the smartphone. The algorithm achieved an accuracy of 98.2% in indoor navigation precision and an accuracy of 0.5 m on average. Jafri et al. [89] used Google Tango Project to serve the visually impaired. The Unity engine’s built-in functions in the Tango SDK were used to build a 3D reconstruction of the local area, and then a Unity collider component was provided to the user, who used it for obstacle detection by determining its relationship with the reconstructed mesh. A method of indoor navigation assistance using an optical head-mounted screen that directs the visually impaired is presented in [66]. The program creates indoor maps by monitoring a sighted person’s activities inside of the facility, develops and prints QR code location markers for locations of interest, and then gives blind users vocal direction. Pare et al. [90] investigated a smartphone-based sensory replacement device that provides navigation direction based on strictly spatial signals in the form of horizontally spatialized sounds. The system employs numerous sensors to identify impediments in front of the user at a distance or to generate a 3D map of the environment and provide audio feedback to the user. A navigation system based on binaural bone-conducted sound was proposed by the authors of [91]. The system performs the following functions to correctly direct the user to their desired point. Initially, the best bone conduction device for use is described, as well as the best contact circumstances between the device and the human skull. Secondly, using the head-related transfer functions (HRTFs) acquired in the airborne sound field, the fundamental performance of the sound localization replicated by the chosen bone conduction device with binaural sounds is validated. A panned sound approach was also approved here, which may accentuate the sound’s location.

iMove Around and Seeing Assistant [92,93] enable users to know their current location, including the street address, receive immediate area details (Open Street Map), manage their points, manage paths, create automated paths, navigate to the selected point or path, and exchange newly generated data with other users. The app can use voice commands to facilitate the control of the program. Seeing Assistant Move has an exploration mode that utilizes a magnetic compass to measure the direction correctly, which transmits this knowledge using clock hours. It also has a light source detector that allows the user to interact with devices that use a diode as an information tool. For people who are fully blind, the light source detector is extremely useful. When leaving the house or planning to sleep, the blind consumer can avoid leaving a lamp turned on. In order to be able to accommodate signaling devices (such as diodes and control lights), the program has a feature that helps a user to detect a blinking light. This can be used to indicate whether or not a device is turned on, or whether or not a battery level is low or high. On the other hand, a great deal of speech, correlated with the user position, is registered by the iMove around app. A note of speech is played any time when the user is near the position where it was captured. BlindExplorer [94] utilizes 3D sounds as auditory stimuli, which offers the app a type of feedback that helps a consumer to travel to the route or destination or in the right direction without needing to visualize the screen and without moving their eyes away from the ground. Right Hear [95] is a virtual access assistant that helps users to easily navigate new environments. It has two modes, including indoor and outdoor. It locates the visually impaired user’s current location and nearby points in indoor and outdoor environments. However, indoor location is limited by supported locations.

Ariadne GPS [96] has a feature that makes the app suitable for the visually impaired. Namely, it uses VoiceOver in the app to inform the user about the street names and numbers that are around them, activated by touch. By simply placing the finger on the device’s screen, the user can be told about the streets while viewing the map and moving it. The user location is in the middle of the screen, and everything in front of the user is in the top half of the screen, while the bottom half of the screen shows that wis behind the user. This app reports on the user’s position at all times. It has a monitor function that works during activation, and it informs the user about their location continuously. BlindSquare [97] is a self-voicing software combined with third-party navigation applications that provides detailed points of interest and intersections for navigating both outside and inside and is designed especially for the visually impaired, blind, deafblind, and partially sighted. To determine what data are most important, BlindSquare has some special algorithms and talks to the user through high-quality speech synthesis. The app can be controlled by voice commands. The Voice Command feature is a paid service that requires credits to be purchased for its continuous use. Voice Command credits are available on the App Store as an in-app purchase.

#### 2.4.3. Facial and Emotion Recognition

Morrison et al. [98] investigated the technological needs of the visually impaired through tactile ideation. Their findings were critical for people with visual disabilities and pointed to the need for social information, such as facial recognition and emotion recognition. In addition, social engagement and the ability to watch what others are doing, as well as the simulation of a variety of visual skills such as object recognition or text recognition, were important abilities. The importance of knowledge of people’s emotions was discussed in [62]. The authors used computer vision technology to solve the problem. Their system uses facial recognition applications to capture six basic emotions. Then, it conveys this emotion to the user by means of a vibration belt. The proposal faced several challenges, involving lighting conditions and the movements of the person opposite the user who was facing the camera directly while the pictures were captured. Therefore, the recognition accuracy was affected. The authors did not present any numerical accuracy information in their paper.

#### 2.4.4. Color and Texture Recognition

Medeiros et al. [65] present a finger-mounted wearable that can recognize colors and visual textures. They developed a wearable device that included a tiny camera and a co-located light source (an LED) that was placed on the finger. This technology allows users to obtain color and texture information by touch, allowing them to glide their fingers across a piece of clothing and combine their understanding of physical materials with automated aural input regarding the visual appeal. The authors used a special camera for this purpose. To identify visual textures, they used a machine learning approach, while color identification was performed through super-pixel segmentation to help with the higher cognition of clothing appearance.

Color Inspector [99] was developed to help blind and other visually disabled people to distinguish color by analyzing live footage in order to explain the color in view and to recognize complicated colors. It supports VoiceOver, which can audibly read out the color. Color Reader [100] allows for the real-time identification of colors solely by pointing the camera at the object. The app has a feature for reading the colors in the Arabic language. ColoredEye [101] provides different color categories with different color descriptions, such as BASIC, with 16 fundamental colors, CRAYOLA, with 134 fun pigments, and DETAILED, with 134 descriptive colors.

#### 2.4.5. Micro-Interactions

Micro-interaction refers to the animations and configuration adjustments that occur when a user interacts with something [102]. There are four stages involved in setting micro-interactions: First, a micro-interaction is started when a trigger is engaged. Second, triggers can be initiated by the user or by the system. Then, a user-initiated trigger requires the user to take action. In a system-initiated trigger, the software recognizes the presence of particular criteria and takes action. When a micro-interaction is initiated, rules dictate what occurs next. Finally, feedback informs individuals about what is happening. Feedback is defined as whatever a user sees, hears, or feels during a micro-interaction. The meta-rules of the micro-interaction are determined by Loops and Modes [103]. A device mounted on a finger was presented by Oh et al. [58] that uses physical gestures to facilitate the micro-interactions of some common and daily applications, such as setting the alarm and finding and opening an app. The authors carried out a survey to study the efficiency of their methods.

#### 2.4.6. Text Recognition

The important questions that we must consider are how to use this feature and how to convey the important information only, rather than all of the information detected in the surrounding area [104]. The blob is a collection of pixels whose intensity is different from the other nearby pixels. Although the MSER (maximally stable external region) can detect the blob faster, the SWT (stroke width transform) algorithm can detect characters in an image with no separate learning process [104]. Shilkrot et al. [105] developed FingerReader, a reading support system for visually impaired people to assist impaired persons in reading printed texts with a real-time response. This gadget is a close-up scanning device that can be worn on the index finger. As a result, the gadget reads the printed text one line at a time and then provides haptic feedback and audible feedback. The Text Extraction Algorithm, which is integrated with Flite Text-To-Speech, was utilized by the authors of [106]. The proposed technique uses a close-up camera to retrieve the printed text. The trimmed curves are then matched with the lines. The 2D histogram ignores the repeated words. The program then defines the words from the characters and transmit them to ORC. As the user continues to scan, the identified words are recorded in a template. As a result, the system keeps a note of those terms in the event of a match. However, when the user deviates from the current line, they receive audible and tactile feedback. Moreover, if the device does not discover any more printed text blocks, the visually impaired receive signals via tactile feedback, informing them of the line’s ending. SeeNSpeak [107] supports VoiceOver and can audibly interpret text from the photographs of books, newspapers, posters, bottles, or any item with text. The app can also use a wide range of target languages to translate the detected text.

#### 2.4.7. Information Services

In the context of location-based services, a beacon is a tiny hardware device that allows data to be transmitted to mobile devices within a certain range. Most apps require that receivers have Bluetooth switched on and that they download the corresponding mobile app and have location services turned on. Moreover, they require that the receiver accepts the sender’s messages. Beacons are frequently mounted on walls or other surfaces in the area as small standalone devices. Beacons can be basic, sending a signal to nearby devices, but they can also be Wi-Fi- and cloud-connected, with memory and processing resources. Some are equipped with temperature and motion sensors [108]. Perakovic et al. [60] used the beacon technology to inform the visually impaired of the required information, such as notifications about possible obstacles, location of an object, and information about a facility or discounts, and to provide navigation in indoor environments.

#### 2.4.8. Braille Display and Printer

Braille technology is an assistive technology that helps blind or visually impaired individuals to perform basic tasks, such as writing, internet searches, braille typing, text processing, chatting, file downloading, recording, electronic mail, song burning, and reading [109]. Major challenges include the high cost of some current braille technologies and the large and heavy format of some braille documents or books with embossed paper. Braille is a representation of the alphabet, numbers, marks of punctuation, and symbols, composed of cells of dots. In a cell, there are 6–8 possible dots, and a single letter, number, or punctuation mark is created by one cell.

Displayer is an electromechanical mechanism for viewing braille characters that commonly utilizes round-tipped pins lifted through holes on a flat surface, which is often called a refreshable braille display or braille terminal. The visually impaired use it instead of a monitor. Via the braille display, they can insert commands and text, and it conveys text and images on the screen to them by modifying or refreshing the braille characters on the keyboard. They can browse the internet, draft documents, and use a computer in general. Up to 80 characters on the screen can be shown on a braille display, which can be updated when the user moves the cursor across the monitor using the command keys, cursor routing keys, or Windows and screen reader controls. Braille displays of 40, 70, or 80 characters are typically available. For most occupations, a 40-character display is appropriate and sufficient.

There are other important devices that can be used by the visually impaired, especially in the case of learning or employment in the office, such as the Refreshable Braille Display and Braille Embosser. A braille printer or a braille embosser is a device that uses solenoids to regulate embossing pins. It extracts data from computing devices and embosses the information on paper in braille. It produces tactile dots on hard paper, rendering written documents that are clear to the blind. Depending on the number of characters depicted, the cost of braille displays varies from USD 3500 to USD 15,000. In 2012, sixty-three studies aimed at finding new ways to developed refreshable braille were conducted by the Transforming Braille Group. Orbit Reader 20 is the result of these studies, with a 20-cell refreshable braille display [110] that currently costs USD 599. However, Orbit Reader 20 is the basic version and has limited braille characters.

Special braille paper is needed for braille embossers, which is heavier and more expensive than standard printer paper. More pages with the same volume of data are required for braille printing compared to a standard printer. They are also sluggish and noisier. Some braille printers are capable of printing single- or double-sided pages. Although embossers are relatively simple to use, they can be messy and can somewhat differ from one device to another in regard to the quality of the finished product [111]. Braille displays range in price from USD 3500 to USD 15,000, depending on the number of characters depicted. The cost of a braille printer is relative to the volume of braille it generates. Low-volume braille printers cost between USD 1800 and USD 5000, whereas high-volume printers cost between USD 10,000 and USD 80,000 [112].

#### 2.4.9. Money Identifier App

Blind people have difficulty determining the value of the banknotes in their possession, and they usually ask and rely on others to discover the value of the banknotes, which exposes them to the risk of theft or fraud. Money identifier apps are a type of software that can recognize the denomination of banknotes used as currencies. The “Cash Reader: Bill Identifier” app [113] can identify a wide range of currencies. However, it requires a monthly payment subscription. MCT Money Reader [114] is another app that can recognize currencies, including Saudi Riyal, with a cost of SR 58.99 for lifetime use. 

#### 2.4.10. Light Detection

The Light Detector [115] translates any natural or artificial light source it encounters into sound. The software locates the light by aiming the mobile camera in its direction. Depending on the strength of the light, the user can notice a greater or lower tone. It is useful for helping the visually impaired to determine if the lights at home are on or off or to if the blinds are drawn by moving the device upwards and downwards.

#### 2.4.11. External Assistance App

Be My Eyes [116] is an app with which blind or visually impaired users can request help from sighted volunteers. Once the first sighted user accepts the request, a live audio and video call is established between the two parties. With a back-camera video call function, the sighted aide can assist the blind or visually impaired. However, the app depends on an insufficient number of volunteers and has privacy issues because the users share videos and personal information during the connection.

#### 2.4.12. Multifunctional App

Sullivan+ (blind, visually impaired, low vision) [117] depends on the camera shots of the mobile and analyzes them using the AI mode, which automatically seeks the top results that match the pictures taken. The app supports the following functions: text recognition, facial recognition, image description, color recognition, light brightness, and a magnifier. However, its capacities for facial and object recognition need further improvements. Visualize-Vision AI [118] makes use of different neural networks and AI to identify pictures and texts. The software is intended to provide visual assistance to the visually disabled, while still providing a developer mode that enables various AIs to be explored. Another app that can identify an object using artificial neural networks was explored in [119].

A visually impaired person can find an object by calling the name of the object, and the app finds it. Seeing Assistance Home [120] allows users to use an electronic lens for partially visually impaired persons, providing color identification, light source detection, and the ability to scan and produce barcodes and QR codes. VocalEyes AI [121] can assist the visually impaired in the following functions: object recognition; reading text; describing environments, label brands, and logos; facial recognition; emotion classification; age recognition; and currency recognition. LetSee [122] has three functions, including money recognition, which supports several currencies but does not support the Saudi Riyal; plastic card recognition; and light measurement tools to help users to locate sources of light, such as spotlights, cameras, or windows. The higher the brightness of the light is, the louder the sound the user hears is. TapTapSee [123] supports object recognition, barcode and QR code reading, auto-focus notification, and Flash toggle. Aipoly Vision [124] provides its service, including full object recognition features, for monthly fees of USD 4.99. The software also has the functions of currency recognition, text reading, color recognition, and light detection. However, the software is only supported by designated iPhone and iPad devices.

#### 2.4.13. VoiceOver

For visually impaired people, voice over is one of the most useful functions. VoiceOver is a gesture-based screen reader. The user can utilize a mobile device even if they cannot see the screen. VoiceOver provides auditory explanations of what is on the screen.

On an iPhone, for example, when the user touches the screen or drags his finger over it, VoiceOver reads out the name of whatever the user touches, including icons and text. To interact with an item, such as a button or link, or to go to another item, the user can use VoiceOver gestures. VoiceOver creates a sound when the user moves to a new screen and then picks and speaks the name of the first item on the screen (typically in the top-left corner). VoiceOver tells the user when the display changes to the landscape or portrait orientation, the screen dims or locks, and what is active on the lock screen when the user turns on their iPhone [125].

The VoiceOver on iOS communicates with the user through a variety of “gestures”, or motions made with one or more fingers on the screen. Many gestures are location sensitive. Sliding one’s finger over the screen, for example, reveals the screen’s visual contents as the finger passes over them. This allows visually impaired users to explore an application’s actual on-screen layout. A person can activate a selected element by double-tapping, similar to double-clicking a mouse, in the same way a sighted user would. VoiceOver can also switch off the display while keeping the touch screen responsive, conserving battery life. This function is called the “Screen Curtain” by Apple [126].

#### 2.4.14. Virtual Assistant Apps (Voice Commands)

Virtual assistants can help the visually impaired through their ability to control their mobile with voice commands. Here, we investigate the three most popular and recent virtual assistants: Siri, Google Assistant, and Bixby. One of the main concerns that we explore is privacy.

##### Siri

Siri is an assistant that uses voice queries and a natural language user interface to respond to queries, make recommendations, and take action by delegating requests to a set of internet services [127]. Siri assists in a series of tasks, such as phone calls; messaging, setting alarms, timers, and reminders; handling device settings; getting directions; scheduling events and reminders; previewing the calendar; running smart homes; making payments; playing music; checking facts; making calculations; and/or translating a phrase into another language [128]. However, some of the functions need to be visualized in order to be completed, because the assistant is designed for sighted people, not for the visually impaired. It needs improvements in order satisfy their needs. Siri provides various languages, including Arabic.

Apple notes that Siri searches and requests are paired with a specific identifier and not an Apple ID; thus, personal information is not stored for sale to advertisers or organizations. Apple declares that users can reset the identifier by turning Siri off and back on at any point, essentially restarting their interaction with Siri, which would erase user data associated with the Siri identifier. The terms state that personal information can only be used by Apple in order to provide or enhance third-party applications for their products, services, and ads. Private data are not exchanged with third parties for marketing purposes of their own. However, for whatever reason, Apple can use, pass, and reveal non-personal information. This means that Apple’s sites, internet platforms, mobile software, email messages, and third-party product ads use monitoring tools to help Apple to better identify customer behavior, inform the business about the areas of its website accessed by the users, and promote and evaluate the efficacy of advertising and searches. Users may see advertisements dependent on other details in third-party applications; however, the Apple ID of a child also receives non-targeted advertisements on such platforms. The terms of Apple state that the protection of all children is a significant priority of Apple. Apple provides parents with the information they need to determine what would be best for their child.

##### Google Assistant

Google Assistant is a virtual assistant that is powered by artificial intelligence created by Google and is mostly available on smartphones and smart home platforms. Google Assistant can be accessed through its website and can be downloaded from the iOS App Store and the Google Play Store. For the sake of privacy, Google Account is designed with on/off data controls, allowing users to choose the privacy settings that suit them. In addition, as technology advances, Google’s terms note that its privacy policies often change, meaning that privacy is still a user-determined individual option. The terms state that Google can use the personal information of users to provide ads to third parties but report that it does not sell the personal information of users to third parties. Moreover, the terms state that Google can show targeted ads to users, but that users can alter their preferences, choose whether their personal information is used to make ads more applicable to them, and turn such advertising services on or off. The terms also state that Google enables particular collaborators to use their cookies or related technology to retrieve information from a user’s account or computer for advertisement and measurement purposes. Google’s rules, however, note that a child’s customized advertising is not displayed, meaning that advertisements are not focused on the information received from a child’s account.

The terms of Google note that all of its services allow users to connect with other trustworthy and untrusted users and exchange information with other people, such as others with whom a user may chat or share content. If a user has a Google Account, their profile name, profile photo, and activities that they carry out on Google, or on third-party applications that are linked to their Google Account, can appear. In addition, information about a child, including their name, photo, email address, and transactions on Google Play, can be exchanged with members of a family using Google Family Link. These terms and conditions note that Google does not gather or use data for advertising purposes in the Google Cloud or G Suite services and that there are no commercials in the G Suite or Google Cloud Platform. Finally, the terms of Google note that it does not send users personalized advertisements on the basis of specific categories, such as religion, race, health, or sexual orientation.

##### Bixby

For Samsung devices, Bixby [129] is an intelligent digital assistant that hears and records according to the user’s desire and works with their favorite applications. Bixby Vision’s scene description function describes what is displayed on the screen. Personalized Bixby allows the assistant to learn the user’s preferences based on their usage in order to facilitate its utility in the future. In addition, Bixby can handle smart devices with voice commands while attaching the apps to SmartThings. The user can change the TV channel or turn the lights on/off.

There is a privacy notice that describes what is recorded and saved. The information that Bixby requires—and it will not work until the user gives their permission—include the username, birthdate, phone number, email, device identifiers, voice commands, health information, and any information that has been provided through the application, such as the user’s interaction with the app. In addition, some of the information can be sent to an external third party to convert the voice command into text. Some of Samsung’s services allow users to communicate with others, and those other users may view information stored or displayed in the user’s account on the social networking service that they are connecting to. In addition, Samsung can use third-party monitoring technology for a range of purposes, such as evaluating the usage of its services and (in combination with cookies) delivering user-relevant content and advertising. Some third parties may serve to advertise or keep track of which advertisements users see, how frequently they see those advertisements, and what users do in response. The terms note that only restricted representatives of Samsung’s Bixby voice service team can access and otherwise process personal data in accordance with their job or contractual duties. Nevertheless, the terms do not reveal how common security measures in the sector, such as encryption, are used to secure sensitive details in transit or at rest [130]. The word Bixby is not easy to pronounce in Arabic. Currently, Bixby does not support the Arabic language.

##### Alexa

Alexa began as a smart speaker equipped with Alexa software, capable of listening to user questions and answering with replies. Over time, more household gadgets were interconnected through Alexa, and they can be operated by smartphones from anywhere. Amazon first built the Alexa platform to work as a digital assistant and entertainment device, but its application and use grew to encompass IoT, online searching, smart office, and smart home features, substantially improving the way that the average person interacts with technology [131]. To make it easier to interact with Alexa, the developers provide a set of tools, APIs, reference solutions, and documentation [132].

### 2.5. Research Gap

We provide a comparison of LidSonic V2.0 with the related works in Table 2. In Column 2, the technologies used in the particular works are mentioned in their respective rows. In Column 3, we discuss the work settings (i.e., whether they were indoor or outdoor). After that, the studies are examined in regard to the capacity for detecting transparent object features. We verify whether the gadget is handsfree. It is critical to know whether the device can operate at night, which is documented in Column 7. We also note whether or not machine learning techniques were used in the research. We also examine the different forms of feedback that they provided and whether they used verbal feedback. In addition, we examine the processing speed, because the solution requires real-time and quick data processing. We also discuss whether the gadget has a low energy consumption. We also explore the device’s cost effectiveness and whether it is inexpensive, and also whether or not the solutions given require low memory, as well as their weights. The various studies relate to, and satisfied the requirements of, some of the system’s essential features. All of these aspects of system design are addressed in our work. To ensure maturity and robustness, further system optimization and assessments are required. A detailed comparison of LidSonic V1.0, which also applies to LidSonic V2.0, is provided in [39].

We noted earlier that, despite the fact that several devices and systems for the visually impaired have been developed in academic and commercial settings, the current devices and systems lack maturity and do not completely fulfil user requirements and satisfaction. We created a low-cost, miniature green device that can be built into or mounted on any pair of glasses or even a wheelchair to assist the visually impaired. Our method allows for faster inference and decision-making while using relatively little energy and smaller data sets. The focus of this paper is the facilitation of the mobility of the visually impaired for the reason that this is one of the most basic and important tasks required for the visually impaired to be self-reliant, as explained in Section 1. The broader literature review was provided in this section to make the reader aware of other requirements of, and solutions for, the visually impaired, to break research barriers, and enable collaboration between different solution providers, leading to the integration of different solutions to create holistic solutions for the visually impaired. Increased and collaborative research activity in this field will encourage the development, commercialization, and widespread acceptance of devices for the visually impaired.

## 3. A High-Level View

In Section 3.1, Section 3.2 and Section 3.3, we present a high-level view of the LidSonic V2.0 system, the user view, the developer view, and the system view. A detailed description of the system design is provided in Section 4.

### 3.1. User View

**Figure 2:**
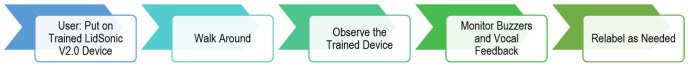
LidSonic: A User’s View.

Figure 2 shows the user view. The user puts on the LidSonic V2.0 gadget, which is fixed in a glass frame. The user installs the LidSonic V2.0 smartphone app after downloading it. Bluetooth connection between the LidSonic V2.0 mobile app and the LidSonic V2.0 device is used. LidSonic V2.0 is intensively trained in both indoor and outdoor settings. The user wanders around in both indoor and outdoor surroundings, allowing the LidSonic V2.0 gadget to be further trained and validated. Furthermore, a visually impaired person’s family member or a volunteer may move around and retrain and check the gadget as needed. The gadget has a warning system in case the user encounters any impediments. When the user encounters an obstacle, a buzzer is activated. Additionally, the system may provide vocal input, such as “Ascending Stairs”, to warn the user of an impending challenge. By pressing the prediction mode screen, the user may also hear the result. A user or his/her assistant can also use voice commands to label or relabel an obstacle class and create a dataset. This enables the validation and refining of the machine learning model, such as the revision of an object’s label in the case that it was incorrectly categorized.

### 3.2. Developer View

**Figure 3:**
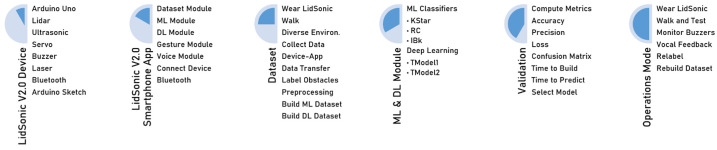
LidSonic: A Developer’s View.

The development of modules, as seen in Figure 3, starts with the construction of the LidSonic V2.0 device. A LiDAR sensor, ultrasonic sensor, servo, buzzer, laser, and Bluetooth are all connected to an Arduino Uno CPU used to build the LidSonic V2.0 gadget. Then, using an Arduino sketch, we combined and handled the different components (sensors and actuators), as well as their communication. The LidSonic V2.0 smartphone app was created with Android Studio (LidSonic V2.0). We created the dataset module to help with the dataset generation. Then, the chosen machine or deep learning module was used to construct and train the models. We utilized the Weka library for the machine learning and the TensorFlow framework for the deep learning models. Bluetooth is used to create a connection between the LidSonic V2.0 device and the mobile app, which is also used to send data between the device and the app. The Google speech-to-text and text-to-speech APIs were used to develop the speech module.

The developer wears the LidSonic V2.0 device and walks around to create the dataset. The LidSonic V2.0 device provides sensor data to the smartphone app, which classifies obstacle data and generates the dataset. To verify our findings, we used standard machine and deep learning performance metrics. The developer wore the trained LidSonic V2.0 gadget and went for a walk to test it in the operational mode. The developer observed the system’s buzzer and vocal feedback. The dataset can be expanded and recreated by the developers, users, or their assistants to increase the device’s accuracy and precision.

### 3.3. System View

**Figure 4:**
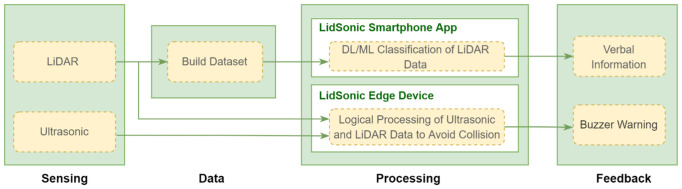
LidSonic V2.0 Overview (Functional).

LidSonic V2.0 detects hazards in the environment using various sensors, analyzes the data using multiple channels, and issues buzzer warnings and vocal information. With the use of an edge device and an app that collects data for recognition, we propose a technique for detecting and recognizing obstacles. Figure 4 presents a high-level functional overview of the system. When the Bluetooth connection is established, the data is collected from the LiDAR and ultrasonic sensors. An obstacle dataset should be established if the system does not already have one. The dataset is created using LiDAR data only. Two distinct channels or procedures are used to process the data. First, simple logic is used by the Arduino unit. The sensors operated by the Arduino Uno controller unit offer the essential data required for visually impaired people to perceive the obstacles surrounding them. It processes the ultrasonic and basic LiDAR data for rapid processing and feedback through a buzzer. The second channel is the use of deep learning or machine learning techniques to analyze the LiDAR data via a smartphone app and produce vocal feedback. These two channels are unrelated to one another. The recognition process employs deep learning and machine learning approaches and is examined and evaluated in the sections below.

**Figure 5:**
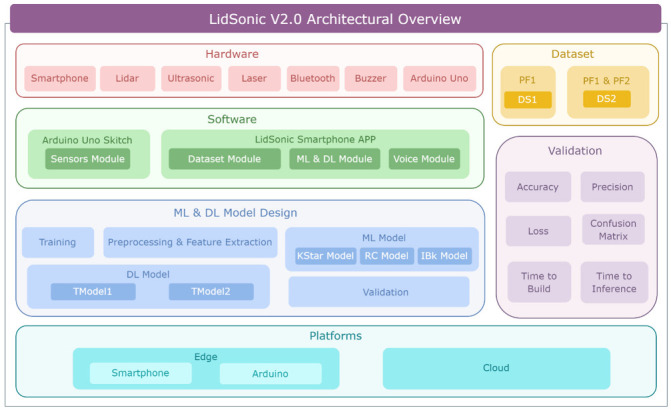
LidSonic V2.0 Overview (Architectural).

Figure 5 depicts a high-level architectural overview of the system. The hardware, machine learning and deep learning models, software, datasets, validation, and the platform are all part of the system. The hardware includes all of the components required by the LidSonic V2.0 gadget. We created several models using ML and DL techniques that are explained further in Section 4. The system makes use of two types of software: one for controlling the sensors and performing the obstacle detection tasks with an Arduino skitch device, and another for the recognition tasks using the smartphone app. The accuracy, precision, loss, time to train a model, time to predict an object, and confusion matrix were employed as validation metrics in this work. Depending on the type and performance of the classifier, the system can be used on a variety of platforms, including edge and cloud. In the next section, we expand this system perspective with comprehensive diagrams and methods.

## 4. Design and Implementation

This section explains the LidSonic V2.0 System’s design in detail. The hardware components and design are described in Section 4.1. Section 4.2 provides an overview of the system’s software design. The sensor module is illustrated in Section 4.3 and the dataset and machine and deep learning modules are explained in Section 4.4 and Section 4.5, respectively.

### 4.1. System Hardware

**Figure 6:**
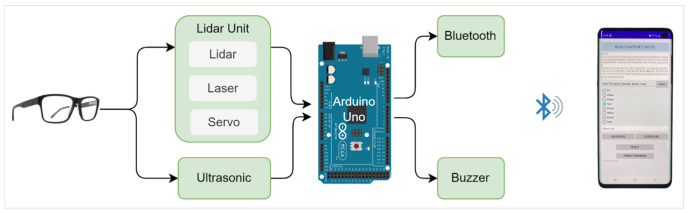
System Hardware.

The system incorporates the following hardware components, as shown in Figure 6: TFmini Plus LiDAR, an ultrasonic sensor, Bluetooth, Arduino Uno, and the user’s smartphone. A servo, buzzer, and power bank are used to operate the device.

**Figure 7:**
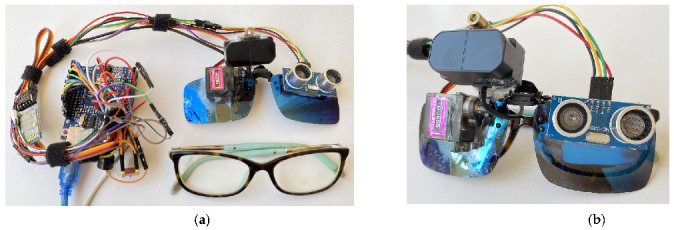
(**a**) LidSonic V2.0 device and a glass (**b**) LidSonic V2.0 device mounted into a glass frame.

Figure 7 displays a photo of the LidSonic V2.0 gadget, which includes smart glasses with sensors and an Arduino Uno board. The LidSonic V2.0 device’s central nervous system is the Arduino Uno microcontroller unit, which is used to integrate and manage the sensors and actuators and to transfer sensor data to the smartphone app through Bluetooth. It is configured so as to control how the servo motions, sensors, and other components interact. The LiDAR Unit contains the TFmini Plus LiDAR sensor [135] that is connected with a servo and laser as a unit. The laser beam is installed above the TFmini Plus LiDAR and helps to indicate where the LiDAR is pointed so that one may scan and categorize various things in order to build a valid dataset. The data collected by the TFmini Plus LiDAR from its spatial environment is transferred to the Arduino unit. Some of this information is used by Arduino to detect obstacles and activate the buzzers as needed, while other information is relayed through Bluetooth to the smartphone app. The servo that controls the movement of the two devices comprises both the TFmini Plus LiDAR and the laser. The ultrasonic sensor is capable of detecting a wide range of obstructions. It is also utilized to compensate for the TFmini’s LiDAR’s inadequacies by recognizing transparent obstructions on the route of visually impaired people. The ultrasonic sensor detects objects at 30 degrees and has a detection range of 0.02 m–4.5 m [136]. The Arduino unit analyzes the data from the ultrasonic sensor, and if an item is detected, the buzzer is actuated. The buzzer sounds with distinct tones to notify visually impaired persons of different sorts of objects detected by the sensors. The buzzer and sound frequencies, or tones, are controlled by the Arduino CPU based on the identified items. A microphone for user instructions, Bluetooth for interfacing with the LidSonic V2.0 device, and speakers for vocal feedback regarding the detected objects are all included in the smartphone app’s hardware.

#### 4.1.1. TFmini-S LiDAR

A laser diode emits a light pulse, which is used in a LiDAR. Light strikes and is reflected by an item. A sensor detects the reflected light and determines the time of flight (ToF). The TF-mini-S device is based on the OPT3101 and is a high-performance, single-point, short-range LiDAR sensor manufactured by Benewake [137]. It is based on long-range proximity and distance sensor analog front end (AFE) technology based on ToF [137]. A TFmini-S LiDAR operates on the networking protocol UART (TTL)/I2C, can be powered by a conventional 5 V supply, and has a total power consumption of 0.6 w.

The TFmini-S LiDAR has a refresh rate of 1000 Hz and a size range of 10 cm to 12 m. It provides a ±6 cm accuracy between 0.1 m and 6 m, and a 1 percent accuracy between 6 m and 12 m. The operational temperature range is from around 0 °C to 60 °C. The range of the angles is 3.5° [138]. Data from the TFmini-S LiDAR may be collected quickly and precisely. There are no geometric distortions in the LiDAR, and it may be utilized at any time of day or night [138]. When no item is identified within a 12 m range, the sensor sends a value of 65,535.

The TFmini-S has the advantages of being inexpensive in cost, having a small volume, low energy consumption, and many interfaces in order to satisfy various requirements, but it has the disadvantage of not being able to detect transparent objects, such as glass doors (we used an ultrasonic sensor to compensate for this). It improves the outdoor efficiency and accuracy with various degrees of reflectivity by detecting stable, accurate, sensitive, and high-frequency ranges. Few studies have been conducted on the utilization of LiDAR to assist the visually impaired and identify their needs. The gadgets that aid the visually impaired make use of a very expensive Linux-based LiDAR [139].

#### 4.1.2. Ultrasonic Sensor

An ultrasonic sensor is one of the best tools for detecting barriers because of its cheap price, low energy consumption, sensitivity to practically all types of artifacts [40], and the fact that the ultrasonic waves may be transmitted up to a distance from 2 cm to 300 cm. Furthermore, ultrasonic sensors can detect items in the dark, dust, smoke, in cases of electromagnetic interference, and tough atmospheres [140].

A transducer, in an ultrasonic sensor, transmits and receives ultrasonic pulses, which carry information about the distance between an item and the sensor. It sends and receives signals using a single ultrasonic unit [41]. The HC SR04 ultrasonic sensor has a <15° effective angle, a resolution of 0.3 cm, a frequency of operation of 40 kHz, and a measurement angle of 30°. The range limit of ultrasonic sensors is reduced when they are reflected off smooth surfaces, when they have a low incidence beam and when they open narrowly. Optical sensors, on the other hand, are unaffected by these issues. Nonetheless, the optical sensors’ shortcomings include the fact that they are sensitive to natural ambient light and rely on the optical properties of the object [37]. Sensors are often employed in industrial systems to calculate object distance and flow velocity. ToF is the time required for an ultrasonic wave to travel from the transmitter to the receiver after being reflected by an object. Equation (1) can be used to calculate the distance from the transmitter, where c is the velocity of the sound [141]:

d=[c ×( ToF)]/2

Infrared sensors and lasers are outperformed by ultrasonic sensors. Infrared sensors cannot work in the dark and produce incorrect findings when there is no light. However, there are inherent drawbacks that restrict the application of ultrasonic instruments to mapping or other jobs requiring great accuracy in enclosed spaces. Due to sonar cross-talk, they are less reliable and have a reduced range, large beam coverage, latency, and update rates [12]. The receiver detects an undetectable small volume of the reflected energy if the obstacle surface is inclined (i.e., surfaces formed of triangles or with rough edges), which causes the ultrasonic range estimations to fail [142].

### 4.2. System Software

**Figure 8:**
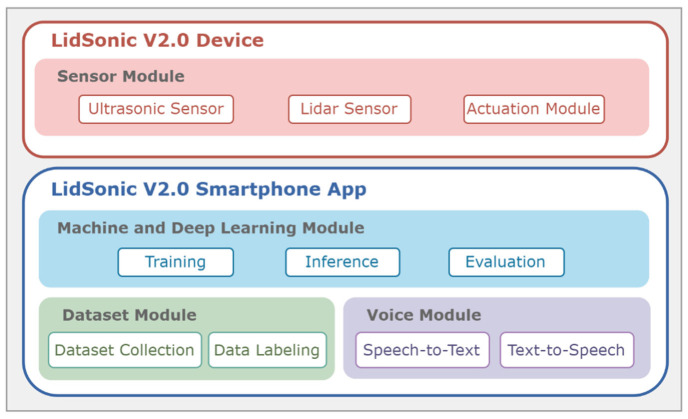
LidSonic V2.0 Software Modules.

The LidSonic V2.0 system consists of the LidSonic V2.0 device and the LidSonic V2.0 Smartphone App (see Figure 8). The LidSonic V2.0 device’s sensor module contains software that controls and manages the sensors (LiDAR and ultrasonic sensors) and actuators (the servo and laser beam). This module also carries out the basic logical processing of sensor data in order to generate buzzer alerts regarding discovered items.

The smartphone app’s dataset module collects data from the LidSonic V2.0 device and appropriately stores the dataset, including the labels. The machine and deep learning module is located in the smartphone app and allows the models to be trained, inferred, and evaluated. Two Google APIs are used by the voice module. The text-to-speech API is used to provide audio feedback from the smartphone app, such as spoken input regarding adjacent objects identified by the sensors, using the mobile speakers. The Google speech-to-text API is used to transform user voice instructions and evaluate them so that the app can take relevant actions, such as labeling and relabeling data objects.

The master algorithm is given in Algorithm 1. The array VoiceCommands (various commands sent to the LidSonic V2.0 System) and AIType are the master algorithm’s inputs. AItype indicates the type of classification approach to be used, either machine Learning (ML) or deep learning (DL). Label, Relabel, VoiceOff, VoiceOn, and Classify are the VoiceCommands. The user gives the system the Label and Relabel voice commands to Label or Relabel an object observed by the system. The commands VoiceOff and VoiceOn are used to switch voice commands on and off if the user simply wants to hear the buzzer sound that alerts them when an object is close rather than hearing the names of all the things being recognized in the surroundings. When the user wants to identify a specific obstacle, they can use the voice command Classify. This command can be used even if the vocal feedback is turned off. The master algorithm produces three outputs: LFalert, HFalert, and VoiceFeedback, which are used to notify the user about various items through a buzzer or voice instruction.
**Algorithm1:** The Master algorithm: LidSonic V2.0**Input:** VoiceCommands (Label, Relabel, VoiceOff, VoiceOn, Classify),     AIType (ML, DL) **Output:** LFalert, HFalert, VoiceFeedback ServoSensorsModuleSubSystem (Angle, Position) LaserSensorsModuleSubSystem ( ) UD2O ← UltrasonicSensorsModuleSubSystem ( ) [LD2O, LDO] ← LiDARSensorsModuleSubSystem ( ) FeedbackType ← ObsDetWarnSensorsModuleSubSystem ( ) **switch** (AIType) **do** **case:** ML   [MLDataset] ← MLDatasetModule (LDO, Label, Relabel)   [MOL, VoiceCommands] ← MLModule (MLDataset, VoiceCommands) **case:** DL   [DLDataset] ← DLDatasetModule (LDO, Label, Relabel)   [DOL, VoiceCommands] ← DLModule (DLDataset, VoiceCommands) **End switch** VoiceModule (VoiceCommands, VoiceFeedback)

The LidSonic V2.0 system operates different modules and subsystems for numerous purposes, as shown by the master algorithm. The ServoSensorsModuleSubSystem, a subsystem of the Sensors module, uses the angle and position as inputs to determine the servo starting position and motion and control the position of the LiDAR sensor. The LaserSensorsModuleSubSystem displays the direction in which the LiDAR is pointing (this is only for development purposes and assists the developer in identifying the object being scanned by the LiDAR). The UltrasonicSensorsModuleSubSystem returns the data output from the ultrasonic sensor, “UD2O” (the user’s distance from the object computed based on the data from the ultrasonic sensor). The LiDARSensorsModuleSubSystem returns two outputs from the LiDAR sensor, “LD2O” (the user’s distance from the object computed based on the data from the LiDAR sensor) and “LDO” (the user’s distance from the object (LiDAR data object that contains detailed data about the objects). The ObsDetWarnSensorsModuleSubSystem returns “FeedbackType”, detects objects, and informs the user about them via buzzers and voice feedback. The MLDatasetModule provides the Weka datasets, labeled “MLDataset”, after receiving the inputs “LDO”, “Label”, and “Relabel”. The DLDatasetModule provides CSV files, the “DLDataset”. The MLModule returns “MOL” (the object level below or above the floor) and “VoiceCommands”. The DLModule returns “DOL” (the object level below or above the floor) and “VoiceCommands”. The VoiceModule transforms speech to text and vice versa using VoiceCommands and VoiceFeedback as inputs. In the next sections, more algorithms, pictures, and text are used to describe the four modules, as well as the inputs and outputs.

### 4.3. Sensor Module

**Figure 9:**
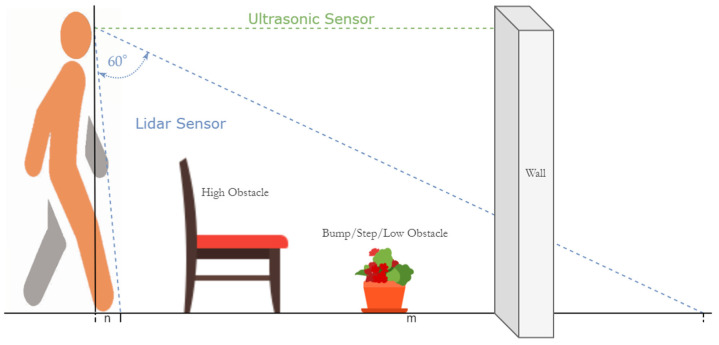
Sensor Coverage.

Figure 9 illustrates how the LidSonic V2.0 pair of glasses use ultrasonic and LiDAR sensors to observe the world. The ultrasonic sound pulse is directed in front of the user, as seen by the dotted green line, to detect any objects in front of the user. It can also detect obstacles that are transparent, such as glass doors or walls, which LiDAR may miss. The LiDAR sensor range is represented by dotted blue lines. The LiDAR sensor has a range of 10 cm to 12 m. We covered a 60-degree region in front of the user with a servo motor that moves the LiDAR sensor, which equates to an area of “m” meters on the floor. This floor area “m”, covered by the LiDAR sensor for a user of 1.7 m in height, would be around 3.5 m. Note that we ignored the fact that the glasses are at eye level rather than head level. The figure also displays the floor area “n”, which is the closest floor area to the user that is not covered by the LiDAR sensor, since we deactivated it in order to minimize false alarms triggered by the user’s knee while walking. This floor space closest to the user would be around 0.15 m for a person of 1.7 m in height. Within this “m” floor space, the LidSonic V2.0 system identifies any obstacles, including descending the stairs, using the LiDAR sensor.

**Figure 10:**
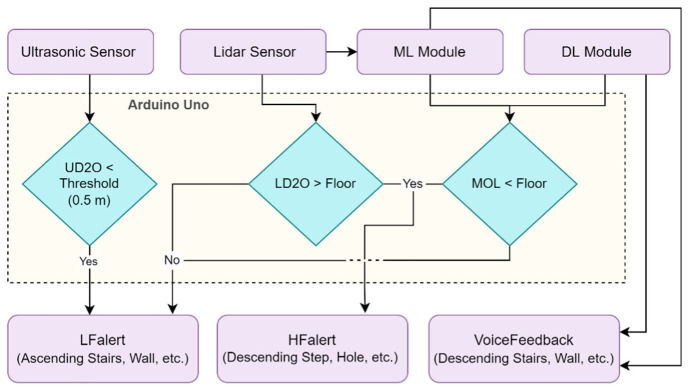
Detection and Warning System.

The flow diagram of the obstacle detection and warning subsystem is shown in Figure 10. The LidSonic V2.0 device uses data from an ultrasonic sensor (UD2O stands for distance to object detected by the ultrasonic sensor) and activates a buzzer with a low-frequency warning, providing an LFalert if it falls below a threshold of 0.5. The LidSonic V2.0 device additionally checks the closest point read by the LiDAR sensor (LD2O is the D2O detected by the LiDAR sensor), and if it is higher than the floor, the LFalert is triggered, signaling that the obstacle might be a high obstacle, a bump, and/or ascending stairs, etc. If it is below the floor, this means that there are obstructions such as descending stairs and/or holes, and a high-frequency alarm HFalert buzzer tone is triggered. The ML module provides the user with voice input depending on the anticipated obstacle type. MOL is converted from the predicted value (object level detected by the ML module). The buzzer is actuated with the LFalert if the predicted value type is an object above the floor; otherwise, the HFalert is triggered. The predicted value is converted to DOL (object level detected by the DL module). If the predicted value type is an object above the floor, the buzzer is activated with the LFalert; otherwise, the HFalert is activated. The figure only shows “MOL < Floor”; however, the values of MOL or DOL are used based on the algorithm used.

Algorithm 2 depicts the algorithm for the obstacle detection, warning, and feedback subsystem. It takes the ultrasonic data (UD2O), nearby LiDAR distance readings (LD2O), the object level calculated by the machine learning module (MOL), and the object level calculated by the deep learning module (DOL) as inputs. The ObsDetWarnSensorsModuleSubSystem function analyzes data for detection and generates audio alarms. High-frequency buzzer tones (HFalert), low-frequency buzzer tones (LFalert), and VoiceFeedback are the output alerts. The subsystem has a logical function that accepts the inputs UD2O, LD2O, and MOL and returns the determination of the kind of obstacle (whether the obstacle is an object above floor level, etc.). No action is required if the output is a floor. If the obstacle returns as HighObs, however, it is either a wall or a high obstacle, and so on.

An LFalert instruction is delivered to the buzzer to start the low-frequency tone buzzer. The buzzer parameter HFalert is used to activate the high-frequency tone buzzer if the obstacle is of the LowObs type. The goal of selecting a high-frequency tone for low obstacle outputs is to ensure that low obstacle outputs are possibly more hazardous and destructive than high obstacle outputs. The high-frequency tone may be more noticeable than the low-frequency tone.
**Algorithm2:** Obstacle Detection, Warning, and Feedback**Input:** UD2O, LD2O, MOL, DOL **Output:** FeedbackType (HFalert, LFalert, VoiceFeedback)  **Function** ObsDetWarnSensorsModuleSubSystem ( ) Obstacle ← Check (UD2O, LD2O, MOL, DOL) **switch** (Obstacle) **do**   **case:** Floor    skip   **case:** HighObs    Buzzer (LFalert)    VoiceModule (VoiceCommands, VoiceFeedback)   **case:** LowObs    Buzzer (HFalert)    VoiceModule (VoiceCommands, VoiceFeedback) **End switch**

### 4.4. Dataset Module

Machine learning is strongly reliant on data. It is the most important factor that enables algorithm training to become possible and to obtain accurate results from the trained models. Our dataset includes a 680-example training set and is formatted using ARFF files for Weka and CSV files for TensorFlow deep learning. The collection provides distance data obtained from the LiDAR equipment by LidSonic V2.0.

Table 3 shows the eight classes in the dataset, the kinds of obstacles that we accounted for, as well as the number of examples/instances of each. Note that the images in the table include both indoor and outdoor conditions. Deep Hole, Ascending Stairs, Descending Stairs, and Wall were trained in indoor environments, while the rest of the objects were trained in outdoor environments. For our smart glasses, we were not especially concerned with the exact specifications of the objects but rather with the broader types of the objects. For example, differentiating between Descending Stairs and a Deep Hole is important, because the former is an object that blind people might wish to use (go down), while they would avoid the Deep Hole. The type of Deep Hole is not important, because they would aim to avoid a deep hole. We trained the machine and deep learning algorithms with the data generated by the LiDAR sensor for the objects and did not program the software with the specification of the objects. Hence, the objects are not defined.

Table 4 shows the preprocessing and feature extraction approaches. PF1 requires that the LidSonic V2.0 device’s upward line scan is set to the same angle index as the downward scan and ends with the class label. PF2 must complete PF1 and then extract just eleven angle readings by dividing the 60 readings by ten along with the last angle distance data (in essence, skipping every five readings, assuming that an object does not exist in this gap and considering that the user is moving, so that this gap is moving too). It also computes the height of the angle nearest to the user, which is the LidSonic V2.0 device’s starting point, as well as the middle angle of the LidSonic V2.0 device’s scan. We need to calculate the distance from the user to the obstacle d2 and the distance from the user to the ground d1 (*y*-axis), the points for both angles, once we have the two height calculations, which are the *x*-axis points. The slope between h1 and h2 can then be calculated. The two heights and the slope are added to the 11-angle distance readings to create the 14-feature dataset, DS2. The 60-angle distance readings are the dataset features of DS1.

**Figure 11:**
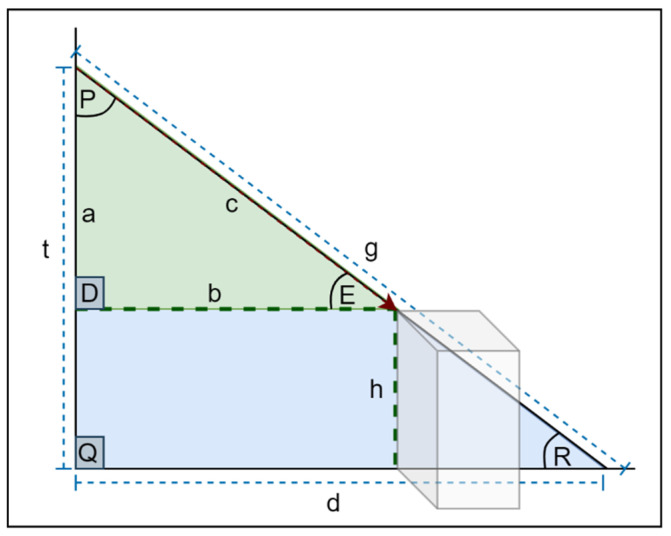
Obstacle Height.

Figure 11 depicts the model used for calculating the obstacle height. The distance between the LidSonic V2.0 device and the ground is represented by g. The larger triangle’s hypotenuse, which is colored blue, is represented by g. The LidSonic V2.0 device’s LiDAR distance from an obstacle is c. We can compute the height of the object h using the similar triangle law and the value of c. Two triangles that have the same ratio of their comparable sides and an identical pair of corresponding angles are called similar triangles.

(1) In ∆PQR and PDE, ∠DPE is common and ∠PDE=∠PQR (corresponding angles). 

(2) ⇒ ∆PQR ~ ∆ PDE (PP criterion for similar triengles)

Hence, from (1) & (2):

(3) ⇒PR/PE=PQ/PD

From Equation (3), calculate r as:

(4) r=c/g

then,

(5) a=t∗r

The height of the obstacle is:

(6) h=t−a

The horizontal distance from the user to the obstacle is calculated by Equation (7):

(7) b=d∗r

There is a ≅ ±3 cm error when computing the height of an object, which we consider insignificant in our case, because we do not require the exact height but, rather, the nature of it (high, low, etc.). The height is calculated and used as a feature in the dataset. We added two height features, h1 and h2, for the purpose of this computation.

**Figure 12:**
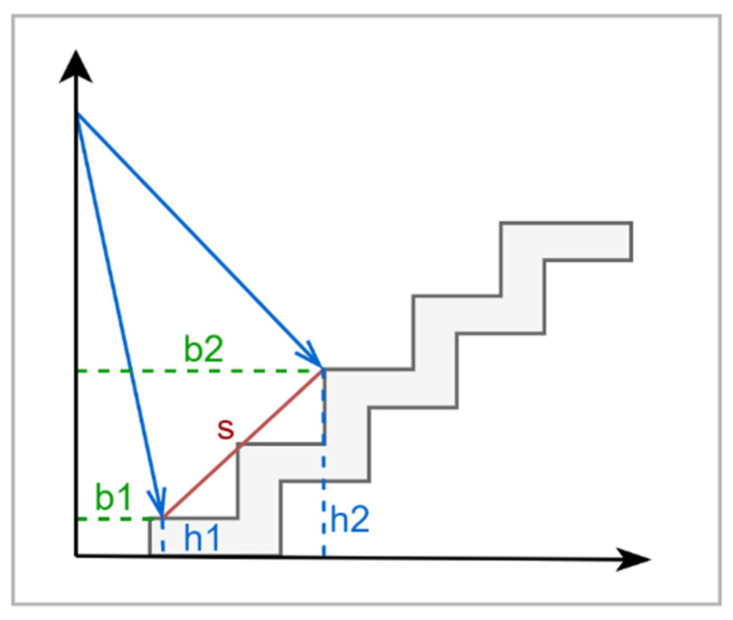
Slope.

Another crucial parameter that we included in our dataset is the slope in Figure 12, between h2 and h1. Since the value of the slope fluctuates depending on the slope of the ground level or the kind of obstacle, especially in the case of stairs, it is a significant factor. Equation (8) calculates the slope as follows:

(8) $$s=\frac{h2-h1}{b2-b1}$$

We created two types of datasets: one that collects 60 values of the features using the LiDAR distance of 60 angles, which we called DS1, and the second dataset, DS2, which extracts 11 features from DS1. Three more features were added: two obstacle heights from two different angles, as well as the slope between these two positions, giving a total of 14 features. We constructed six training models to be examined, evaluated, and analyzed for optimal utilization. Two different approaches were investigated: Weka-based machine learning and TensorFlow-based neural networks. We used K* (KStar), Random Committee, and IBk as classifiers in Weka in order to train six machine learning models. For our system, these were the most successful classification methods [39]. The second is a TensorFlow-based deep learning technique that utilizes the two datasets. We constructed two deep learning models for each. Machine and deep learning algorithms are discussed in the next subsection. The DS1 labels range from a01–a60 and end with the obstacle class to give a total of 61 features. DS2 has 11 angle labels in addition to h1, h2, s, and the obstacle class. The DS1 labels range from a01–a60 and end with the obstacle class to give a total of 61 features. DS2 has 11 angle labels in addition to h1, h2, s, and the obstacle class.

Table 5 and Table 6 list the two types of datasets that we used in our research, along with a sample of collected data for each obstacle class. The DS1 labels range from a01–a60 and end with the obstacle class to give a total of 61 features. DS2 has 11 angle labels in addition to h1, h2, s, and the obstacle class.

Algorithm 3 outlines how our system’s dataset is created. It takes the CSV Header, LDO, and Features as inputs. Using the Building Dataset function, the CSV header file from CSVHeader is first placed in the new dataset, CSVFile. Data are collected from LDO and saved in a LogFile using the DataCollection method. LDO represents the LiDAR distance readings, while the loop records the data in the proper format, saving the LiDAR downwards data in the original order and reversing the order of the LiDAR upward data.
**Algorithm3**: DatasetModule: Building Dataset Algorithm**Input:** Header, LDO **Output:** Dataset  **Function:** BuildingDataset ( )  Insert Header into the File  LogFile ← LDO  **While** (not end of LogFile) //Bluetooth incoming data stored in LogFile   strLine ← BufferLine //BufferLine is a line taken from LogFile   mutualFlag ← true   **While** (strLine ! = 0)    **If** (mutualFlag)     myData ← strLine + Obstacle class     Write myData in the File     Clear myData     mutu-alFlag ← false    **Else**     Store numbers of strLine into an array called strarr     **For** (x = (strarr.length)—1; x ≥ 0; x--)      reverseStr ← reverseStr + strarr[x] + “,”     **End For**     myData ← myData + reverseStr + Obstacle class     Write myData in the File     Clear my-Data and reverseStr    **End If**   **End While**  **End While**


**Figure 13:**
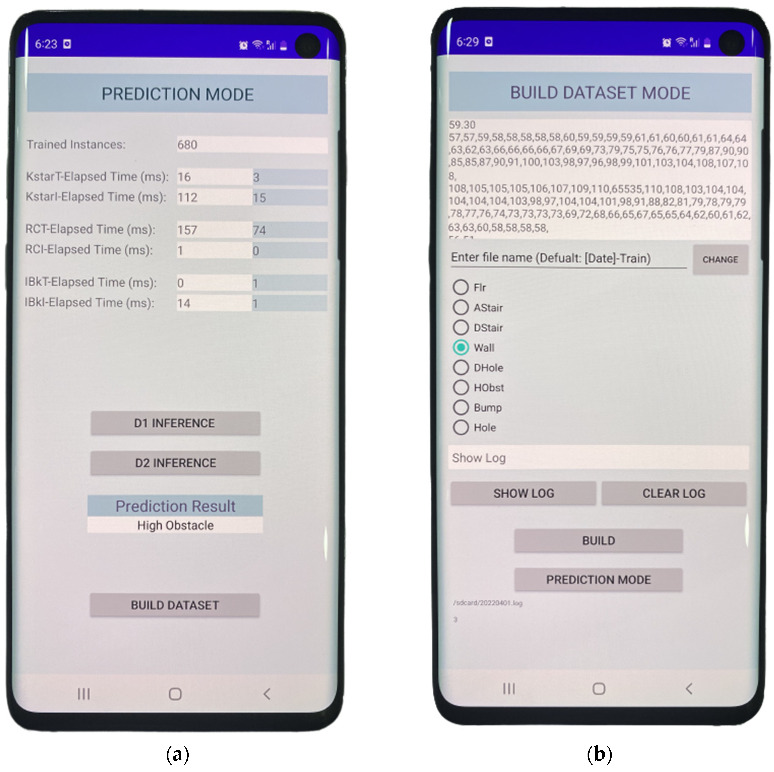
LidSonic V2.0 App. (**a**) Prediction Mode. (**b**) Build Dataset Mode.

Figure 13 depicts the LidSonic V2.0 Smartphone app’s user interface, which is used for building the dataset. The LiDAR sensor sends data to the mobile app through Bluetooth, which it saves in a file named LogFile. On the left-hand side is the prediction mode in which the user hears the verbal feedback of the recognized hazard. In addition, it shows some of the evaluation measurements that are conducted for the three classifiers. For example, KstarT-Elapsed Time (ms) shows the time in milliseconds that is required to build the Kstar classifier (we provide more details regarding the classifiers in the next subsection) for the DS1 dataset in the white box and the DS2 dataset in the blue box. KstarI-Elapsed Time (ms) shows the inference time required to predict an object for datasets DS1 and DS2, respectively. When the D1 INFERENCE button is pressed, the evaluation measurement of DS1 is presented in the white boxes for each classifier, while the D2 INFERENCE button shows the results obtained for DS2.

The LogFile data are displayed in the figure’s right-hand mobile app view. The real number ultrasonic sensor measurements is shown in the first line. We will explore this further in future work to determine whether it is worthwhile to include it as a feature. The ultrasonic measurements were not included in the dataset for this study. The LiDAR sensor’s downward and upward 60-degree readings are presented in the first two lines, which include 60 comma-separated numbers. The LiDAR sensor is linked to a servo that rotates in one-degree increments downwards and upwards, capturing the distance from the device to the object at each degree position. The 60-degree downward and upward measurements are acquired in this manner. Each line of data basically provides 60 measurements of the distance from the user’s eye to the object, each with a distinct line of sight angle. Every two lines of the 60-degree downward and upward measures are followed by a real number, and so on.

### 4.5. Machine and Deep Learning Module

We tested multiple types of models on different types of datasets to determine which one performed the best. Algorithm 4 shows the method used to preprocess the data and extract features. The inputs are Dataset, LIndex1, and LIndex2. This is the method we use to preprocess and extract features from the dataset and the variation between the ARFF files (for WEKA) and CSV files (for TensorFlow) based on the file format. To begin, we proceed to the locations where the dataset’s data begin. The SelectedFeatures function extracts 11 data values from 60 values for each line. The CalculateHeight function takes the two pointing locations of the Lidar sensor, LIndex1 and LIndex2, to acquire their heights, yielding Height [1] and Height [2]. The two numbers are then sent to the CalculateSlope function to determine the slope, finally incorporating the findings into the file.
**Algorithm4:** PreProFXModuleDL: Preprocessing and Feature Extraction**Input:** Dataset, LIndex1, LIndex2 **Output:** Dataset  Go to the first instance **While** (not end of File)  strLine ← BufferLine //BufferLine is a line taken from the Dataset  **While** (strLine ! = 0)   SData [1, …, 11] ← SelectedFeatures()   Height [1], Height [2] ← CalculateHeight (LIndex1,LIndex2)//Equations (4)–(6)   Slope ← CalculateSlope(Height [1], Height [2])//Equations (7) and (8)   DataLine ← AddFeatures(SData[], Height[], Slope)   Write DataLine in the File   Clear DataLine  **EndWhile**
 **EndWhile**

Algorithms 5 and 6 provide high-level algorithms for the machine and deep learning modules. A detailed explanation is presented in Section 4.5.1 and Section 4.5.2.
**Algorithm 5:** Machine learning Module**Input:** Dataset, PrFx **Output:** MOL, VoiceCommands  **If** (PrFx)  Dataset ← PreProFXModuleML(Dataset) **End If** MLModel ← Train (Dataset) [MOL, VoiceCommands] ← Inference (MLModel)

**Algorithm 6:** Deep Learning Module**Input:** Dataset, PrFx **Output:** DOL, VoiceCommands  **If** (PrFx)  Dataset ← PreProFXModuleDL(DLDataset) **End If** DLModel ← Train (Dataset) [DOL, VoiceCommands] ← Inference (DLModel)

The prediction mode can be used to aid visually impaired users in three distinct ways: the prediction button, throw gesture, or voice instruction. To utilize the voice instruction, the user must double-tap the screen so as to access the speech-to-text API and then speak “Prediction Mode” on the command line. The prediction mode is accessed by flinging the screen.

#### 4.5.1. Machine Learning Models (WEKA)

WEKA, a java-based open source program that contains a collection of machine learning algorithms for data mining applications [143], was employed to train the dataset with three classifiers that were carefully selected from detailed experiments carried out in our previous works, which provided the best results, including KStar, IBk, and Random Committee [39].

##### KStar Algorithm

KStar is an instance-based classifier, which means that the class of a test instance is decided by the class of related training examples, as defined by a certain similarity function. It utilizes an entropy-based distance function, which sets it apart from other instance-based learners. Instance-based learners use a dataset of pre-classified examples to categorize an instance. The essential hypothesis is that comparable instances are classified similarly. The issue is that we must determine how to define the terms “similar instance” and “similar class”. The distance function, which defines how similar two examples are, and the classification function, which describes how the instance similarity creates a final classification for the new instance, are the related components of an instance-based learner. The KStar algorithm employs an entropic measure, which is based on the chance of random selection from among all the conceivable transformations, turning one instance into another. It is particularly helpful to use entropy as a metric for the instance distance, and information theory aids in the determining the distance between the instances [144]. The distance between instances determines the complexity of a transition from one instance to another. This is accomplished in two stages. To begin, a limited set of transformations are created that transfer one instance to another. Then, using the program, we can convert one instance from x to y in a limited sequence of transformations that begins with x and ends with y.

##### Instance-Based Learner (IBk) Algorithm

An ideal description is found in the principal output of IBk algorithms (or concept). This is a function that maps instances to create categories. It returns a classification for an instance chosen from the instance space, which is the anticipated value for the instance’s category attribute. A collection of stored examples and, possibly, some information about their historical performances during classifying are included in an instance-based concept description (e.g., their number of correct and incorrect classification predictions). After each training instance is handled, this list of instances may vary. IBk algorithms, on the other hand, do not generate extensive idea descriptions. Instead, the IBk algorithm’s chosen similarity and classification functions determine the concept descriptions based on the current collection of stored instances. The framework that describes all IBk algorithms comprises three sections: the Similarity Function, Classification Function, and Concept Description Updater. These are explained as follows. (1) The Similarity Function determines how similar a training instance x is to the concept description’s examples. Similarities are given as numerical values. (2) The Classification Function takes the results of the similarity function and the classification performance records of the instances in the concept description and uses them to classify them. This leads to an x classification. (3) The Updater for Concept Descriptions is a program that keeps track of the results of the classification and decides which instances should be included in the concept description. Include ‘*I*’ inputs, the similarity outcomes, the classifying results, and the current concept description are all inputs. This results in an updated concept description.

Unlike most other supervised learning approaches, IBk algorithms do not create explicit abstractions, such as decision trees or rules. When cases are provided, most learning methods produce generalizations from these cases and utilize simple matching processes to classify subsequent instances. At the time of presentation, this includes the objective of the generalizations. Because IBk algorithms do not store explicit generalizations, they perform less work at the presentation time. However, when they are supplied with more cases for classification, their workload increases, as they compute the similarities of their previously saved instances with the newly presented instance. This eliminates the need for IBk algorithms to keep rigid generalizations in concept descriptions, which may incur significant costs for their updating in order to account for prediction errors [145].

##### Random Committee Algorithm

The Random Committee algorithm is an ensemble of randomizable base classifiers that may be built using this class. A distinct random number seed is used to build each base classifier (but each is based on the same data). The final prediction is an arithmetic mean of the predictions made by each of the base classifiers [146].

**Figure 14:**
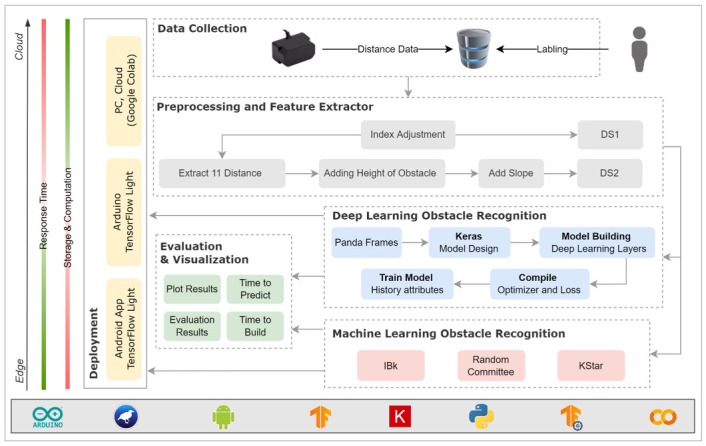
Machine and Deep Learning Modules.

Figure 14 shows the model procedure using the Weka and TensorFlow frameworks. The data are obtained from the LidSonic V2.0 gadget via its sensors and labeled by the user. Next, using the preprocessing and extraction module, we can produce two datasets, DS1 and DS2. Then, these datasets are trained using three machine-learning methods, IBk, Random Committee, and Kstar, in the machine learning obstacle recognition module. In the evaluation and visualization module, six Weka models were evaluated using three classifiers and two datasets. We used a 10-fold cross-validation to evaluate the training datasets. Weka runs the learning algorithm eleven times in the 10-fold cross-validation, once for each fold of the cross-validation and once more for the complete dataset. Each fit is performed using a training set made up of 90% of the entire training set, chosen at random, with the remaining 10% utilized as a hold-out set for validation. The deployment may be performed in a variety of ways, and we chose the optimal deployment method on the basis of the performance and analysis of each classifier.

**Figure 15:**
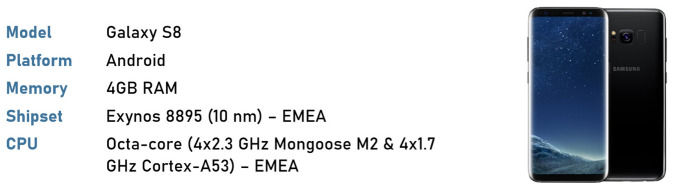
Samsung Galaxy S8 Specification.

The training model building time was evaluated on a Samsung Galaxy S8 mobile (see Figure 15). The mobile has 4 GB RAM; Exynos 8895 (10 nm), EMEA shipset; and Octa-core (4 × 2.3 GHz Mongoose M2 & 4 × 1.7 GHz Cortex-A53), EMEA CPU. In the results section, the results are fully clarified.

#### 4.5.2. Deep Learning Models: TensorFlow

The model procedure of the TensorFlow framework is depicted in Figure 14. The user labels the data that is acquired from the LidSonic V2.0 device via its sensors. After that step, we created two datasets, DS1, and DS2 (these are the same datasets that are used for the machine learning models). We built two models, TModel1 and TModel2, to evaluate the two datasets we constructed. The deep models used are convolutional neural networks (CNNs). In the evaluation and visualization module, the models were evaluated, and the results were plotted and analyzed.

The datasets were divided into three sections: training, validation, and testing (see Table 7). The validation set was used to evaluate the loss and any metrics during the model fitting; however, the model was not fitted using this data. In the deployment phase, we put the module into production so that users would be able to make predictions with it. TensorFlow has great capabilities and offers a variety of choices regarding the models to be deployed, including TensorFlow Serving, TensorFlow Light (TinyML), and more. TensorFlow Serving is a TensorFlow library that enables models to be served through HTTP/REST or gRPC/Protocol Buffers. TensorFlow Serving is a model deployment strategy used for machine learning and deep learning models that are flexible and have a high performance. TensorFlow Serving makes it simple to deploy models. TensorFlow Lite is a lightweight TensorFlow solution for mobile and embedded devices that focuses on running machine learning (mostly deep learning) algorithms directly on edge devices, such as Android and iOS, as well as embedded systems, such as Arduino Uno. Tiny machine learning refers to a branch of machine learning microcontrollers and mobile phones. Because most of these devices are low powered, the algorithms must be carefully tuned so as to operate on them. TinyML has become one of the fastest developing subjects in deep learning due to the ability to perform machine learning directly on edge devices and the ease that comes with it. The smartphone, or Arduino Uno microprocessor, in our scenario, is an edge device that employs the final output of machine learning algorithms. Many operators run machine learning models on more capable devices and then send the results to edge devices. This method is starting to change because of the emergence of TinyML.

The datasets are divided as shown in Table 7. During the training phase, the test set is ignored, and it is only utilized at the end in order to assess how well the model generalizes to new data. This is especially essential in the case of unbalanced datasets, when the absence of training data poses a considerable risk of overfitting.

##### ReLU

The model was fine-tuned with layers to increase its accuracy and precision. We employed three layers for the Deep Neural Network in addition to the input and output layers, as shown in Table 8 for DS1 and Table 9 for DS2, and applied the Rectified Linear Unit activation function (ReLU).

##### Softmax Regression

Since we had a multi-class dataset, we employed softmax regression. Softmax regression (also known as multinomial logistic regression) is a generalization of logistic regression used for dealing with several classes. As a result, the softmax function performs two tasks: First, it converts all of the scores into probabilities. Then, the total probability equals 1. The sigmoid function is used for the same problem in the binary logistic classifier to classify two classes. The softmax function is little more than a generalized sigmoid function.

##### Cost Function

We must create a cost function, with which the softmax probability and one-hot encoded target vector must be compared to determine the similarity. For this purpose, we employed the conception of cross-entropy. Cross-entropy is a distance computation function that uses the softmax function’s estimated probability and the one-hot-encoding matrix to determine the distance. The distance values for the correct target classes are lower, while the distance values for the incorrect target classes are greater. Passing an input through the model and comparing the predictions to ground-truth labels are the means by which a neural network is trained. A loss function is used to make this comparison. Categorical cross-entropy loss is the loss function of choice for multiclass classification issues. It does, however, necessitate the one-hot encoding of the labels. Sparse categorical cross-entropy loss may be a useful option in this instance. The loss function offers the same type of loss as categorical cross-entropy loss but on integer targets rather than one-hot encoded targets. This eliminates the categorical step that is so prevalent in TensorFlow/Keras models. In artificial neural networks, the softmax function is employed in a variety of multiclass classification algorithms. The outcome of K’s unique linear functions is used as the input for the multinomial logistic regression and linear discriminant analysis, and the predicted probability is calculated by Equation (9):

(9) $$P\left(y=j|x\right)=\frac{e^{x^{T}w_{j}}}{\sum_{k=1}^{K}e^{x^{T}w_{k}}}$$

with the jth class given as the x sample vector and w weighting vector.

##### Adam Optimizer

Next, we employed Adam as an optimizer. Adam describes the phrase “adaptive moment estimation”. Adam is an optimization algorithm that may be used to update network weights iteratively based on training data instead of the traditional stochastic gradient descent procedure. We can note the following advantages of employing Adam for non-convex optimization problems. Its implementation is simple. It is effective in terms of computation. There are not that many memory demands. The gradients are invariant in regard to diagonal rescaling. It is ideally suited to issues involving a large number of data and/or parameters. It is a great option for non-stationary objectives. It is suitable for gradients that are exceedingly noisy or sparse. Finally, the hyper-parameters are easy to read and usually do not require much adjustment. Adam is a stochastic gradient descent extension that combines the benefits of two earlier extensions, the Adaptive Gradient Algorithm (AdaGrad) and Root Mean Square Propagation (RMSProp). Adam is a popular deep learning method, since it produces good results swiftly. The results were plotted and reviewed during the evaluation and visualization phase. The model configuration of the experiments is shown in Table 10.

### 4.6. Voice Module

A multiplicity of application programming interfaces (APIs) are now accessible for a variety of activities that formerly required significant programming effort on behalf of developers. When working with audio file data, the job becomes more challenging. As a result, we relied on Google’s speech-to-text engine [147], which can transcribe any audio while maintaining the context and language. The API supports up to 120 languages. Other functions include voice command and control, call center audio transcription, real-time streaming, pre-recorded audio processing, and others. The Google speech-to-text tool can successfully translate written text into grammatically and contextually relevant speech using a range of natural voices. The Google text-to-speech API enables developers to interact with customers through speech user interfaces in devices and applications and customize the communication depending on voice and language preferences.

The Voice Module, for example, allows the user to generate the dataset and transition between different development and operation phases using voice commands. To begin the process of producing a dataset, the user types the command “Train”. The system then asks the user “what is the obstacle class?” in order to classify the incoming data. The user specifies the obstacle, such as “Wall”. The system then requests that the user to “Specify the dataset file name”. Finally, the user enters the file name verbally.

## 5. Performance Evaluation

We now analyze the performance of the LidSonic V2.0 system: Section 5.1 discusses the performance using the machine learning models and Section 5.2 discusses the system performance using the deep learning models.

### 5.1. Machine-Learning-Based Performance

There are several metrics defined in the Weka software that can be computed by the model and are useful for measuring the performance. Accuracy is defined as the percentage of properly classified instances. Precision is defined as the percentage of expected positives that were correctly classified. Table 11 displays the accuracy and precision of the six machine learning models, adopting three classifiers to construct models from the two datasets, DS1 and DS2.

**Figure 16:**
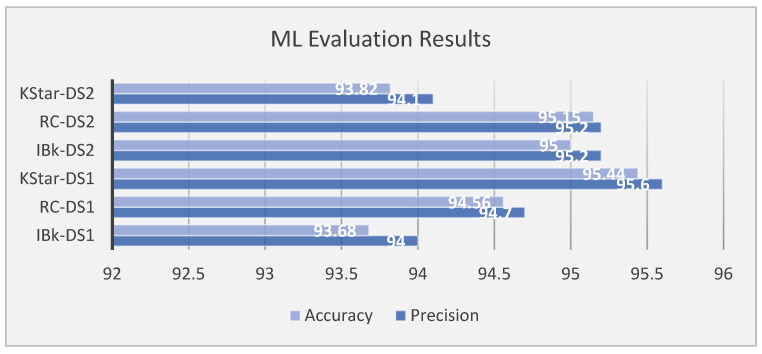
Weka Classifier Evaluation Results.

The results are depicted in Figure 16. The results indicate that using DS2 with Random Committee and IBk classifiers increases accuracy to (95%) and (95.15%), respectively, with equal precision results of (95.2%). The KStar classifier, on the other hand, has greater accuracy (95.44%) and precision (95.6%) when utilizing DS1.

**Figure 17:**
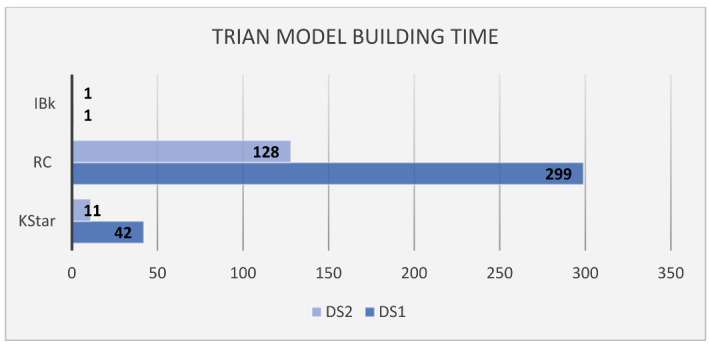
Time Required to Build a Model for each Classifier.

**Figure 18:**
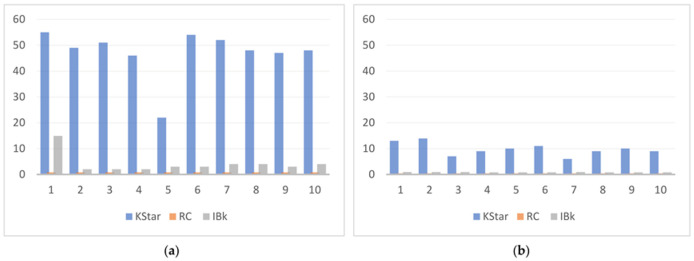
Classifiers’ Inference Elapsed Time in Milliseconds for (**a**) DS1 Test Samples and (**b**) DS2 Test Samples.

Figure 17 plots the model training times required for the top three classifiers. The longest time was spent building the classification model using DS1, compared with the time spent building the classifiers using DS2. Using DS1, RC required the longest time for building (299 ms), followed by KStar (42 ms) and IBk (1 ms). For DS2, RC required the longest time for building (128 ms), followed by KStar (11 ms) and then IBk (1 ms). While RC classifier requires the longest time to build its model, it requires the shortest time to predict an object (see Figure 18).

Figure 18 plots the model inference times for the top three classifiers. For both the DS1 and DS2 test samples, we constructed 10 test predictions and timed each classifier in order to produce the outcome. The time required for KStar to forecast an object varied between 22 and 55 milliseconds when using the DS1 test samples and between 9 and 13 milliseconds when using the DS2 test samples. The IBk classifier, on the other hand, showed faster times than KStar, with an average of 4–5 milliseconds for the DS1 test samples and 0–1 milliseconds for the DS2 test samples. Random Committee required a substantial amount of time to develop its training model, and it predicted the test samples in less than 1 millisecond for both the DS1 and DS2 test samples.

It is worth mentioning that the IBk and KStar algorithms trained and generated models faster than the Random Committee algorithm (see Figure 17). Random Committee required 299 milliseconds to create its trained model using DS1, whereas for DS2 it required 128 milliseconds. KStar additionally required a long time to construct its training model, taking 42 ms for DS1 and 11 ms for DS2. On the other hand, for both DS1 and DS2,the IBk classifier built the trained model in 1 ms. As a consequence, we suggest that the IBk classifier is preferable over the Random Committee and KStar classifiers for the purpose of mobile adaption, embedded microprocessors, and/or large datasets. Although there is a modest accuracy trade-off, the IBk classifier delivers a significant decrease in the mobile computing and battery usage.

When approaching the design of a system architecture that takes advantage of fog or cloud in the training phase, we suggest the KStar classifier, especially in the case of a larger training dataset that will be trained in the layers of fog or cloud, because it can exploit its computation processing powers and return results for the edge level. In the case of the RC classifier, it can be trained in the higher layers (cloud or fog) and the constructed model can then transfer to the edge, because its prediction time is the shortest among the three classifiers.

**Figure 19:**
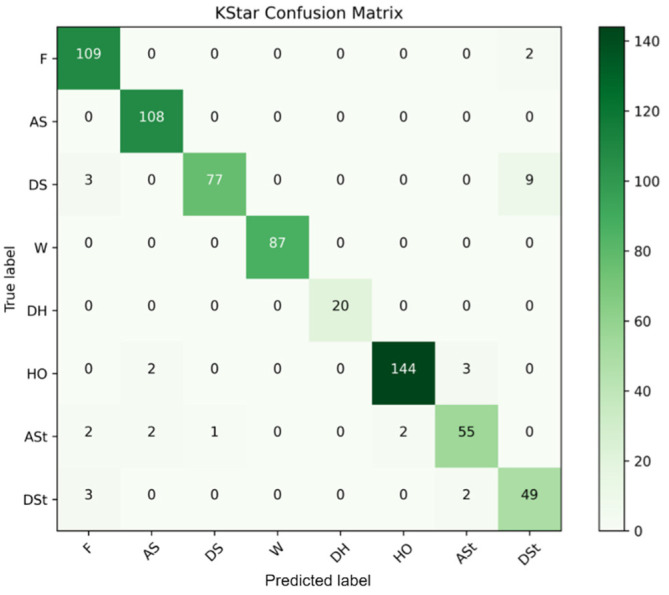
KStar Confusion Matrix using DS1.

**Figure 20:**
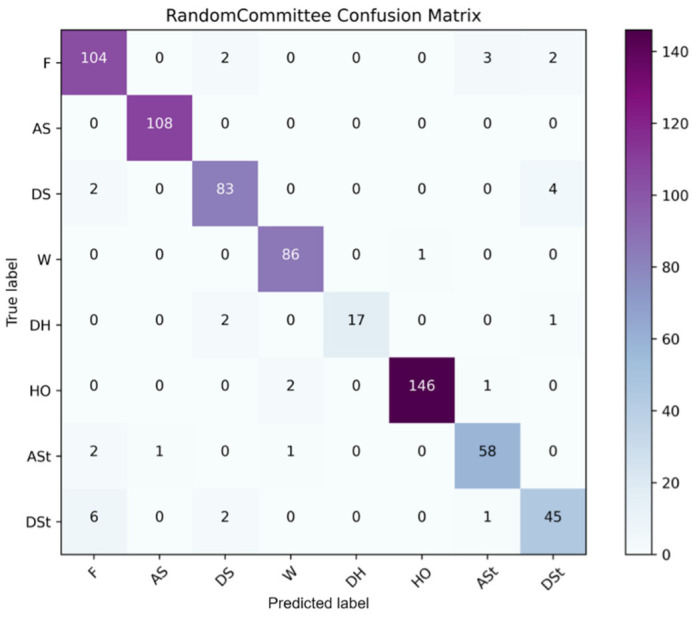
Random Committee Confusion Matrix Using DS2.

**Figure 21:**
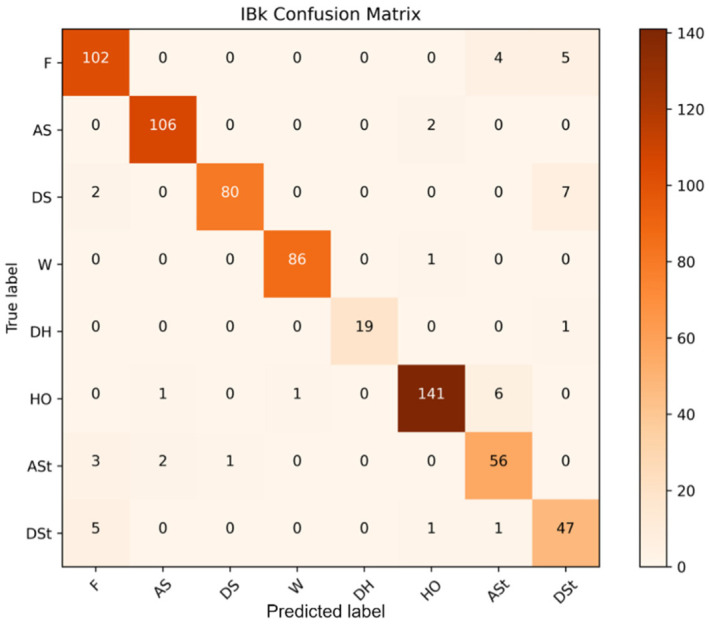
IBk Confusion Matrix Using DS2.

Figure 19 depicts the confusion matrix with the best training model score, which was obtained using the KStar classification method with the D1 dataset. Figure 20 shows the confusion matrix of the Random Committee classifiers trained on DS2. Figure 21 shows the confusion matrix for the IBk classifier. Because these were the top three highest performing classifiers identified in our previous study, we chose to exhibit the confusion matrices of these three classifiers. The abbreviations used in the figures are listed in Table 12.

Figure 19 plots the confusion matrix of the KStar classifier. The highest number of true positives was obtained for the Floor class (109), followed by Ascending Stairs (108), Descending Stairs (77), Wall (87), Deep Hole (20), High Obstacle (144), Ascending Step (55), and Descending Step (49). The total number of wrong predictions for the classes are Floor (2), Ascending Stairs (0), Descending Stairs (3), Wall (0), Deep Hole (0), High Obstacle (5), Ascending Step (7), and Descending Step (5). Obviously, the number of wrong predictions should be considered relative to the total number of instances. It is possible that the higher number of wrong predictions for some classes is due to the low number of instances of the data objects for those classes. Note also that the Floor was misclassified two times as Descending Step. Descending Stairs were misclassified 3 times as Floor and 9 times as Descending Step. The High Obstacle class was misclassified 2 times as Ascending Stairs and 3 times as Ascending Step. Ascending Step was misclassified 2 times as Floor, 2 times as Ascending Stairs, 1 time as Descending Stairs, and 2 times as High Obstacle. Descending Step was misclassified 3 times as Floor and 2 times as Ascending Step. On the other hand, Ascending Stairs, Wall, and Deep Hole classes had no misclassified results.

Figure 20 plots the confusion matrix for the Random Committee (RC) classifier. The highest number of true positives was obtained for the High Obstacle (146) and Ascending Stairs (108) classes, followed by Floor (104), Wall (86), Descending Stairs (83), Ascending Step (58), Descending Step (45), and Deep Hole (17). The total number of wrong predictions for the classes are Floor (7), Ascending Stairs (0), Descending Stairs (6), Wall (1), Deep Hole (3), High Obstacle (3), Ascending Step (4), and Descending Step (9). Note that Floor was misclassified 2 times as Descending Stairs, 3 times as Ascending Step, and 2 times as Descending Step. Descending Stairs were misclassified 2 times as Floor and 4 times as Descending Step. The Wall class was misclassified 1 time as High Obstacle. Deep Hole was misclassified 2 times as Descending Stairs and 1 time as Descending Step. The High Obstacle class was misclassified 2 times as Wall and 1 time as Ascending Step. Ascending Step was misclassified 2 times as Floor, 1 time as Ascending Stairs, and 1 time as Wall. Descending Step was misclassified 6 times as Floor, 2 times as Descending Stairs, and 1 time as Ascending Step. Ascending Stairs had no misclassifications.

Figure 21 plots the confusion matrix for the IBk classifier. The highest number of true positives was obtained for the High Obstacle (141) and Ascending Stairs (106) classes, followed by Floor (102), Wall (86), Descending Stairs (80), Ascending Step (56), Descending Step (47), and Deep Hole (19). The total number of misclassifications for the classes were Floor (9), Ascending Stairs (2), Descending Stairs (9), Wall (1), Deep Hole (1), High Obstacle (8), Ascending Step (6), and Descending Step (7). The greater numbers of incorrect predictions for some classes are likely due to the low number of instances of the data objects for those classes, as we saw with the KStar classifier. Note that Floor was misclassified 4 times as Ascending Step and 5 times as Descending Step. Ascending Stairs were misclassified 2 times as High Obstacles. The Descending Stairs class was misclassified 2 times as Floor and 7 times as Descending Step. The Wall class was misclassified 1 time as High Object and zero times as any other class. Deep Hole was misclassified 1 time as Descending Step and zero times as any other class. The High Obstacle class was misclassified 1 time as Ascending Stairs, 1 time as Wall, and 6 times Ascending Step. Ascending Step was misclassified 3 times as Floor, 2 times as Ascending stairs, and 1 time as Descending Stairs. Descending Step was misclassified 5 times as Floor, 1 time as High Obstacle, and 1 time as Ascending Step. Wall and Deep Hole had the least number of misclassifications.

### 5.2. Deep-Learning-Based Performance

Observing the performance of neural networks and deep learning models over time during training helps to provide researchers with knowledge about them. Keras is a Python framework that encapsulates the more technical TensorFlow backends and provides a clear interface for generating deep learning models. We used Keras in Python to evaluate and display the performance of deep learning models over time during training so as to measure their accuracy and loss. Note that, here, the deep learning models were trained and executed on a laptop device. Future work will attempt to implement deep learning models on mobile phones and other edge devices using TFLite, as in our other strands of research [148]. Table 13 summarizes the findings.

**Figure 22:**
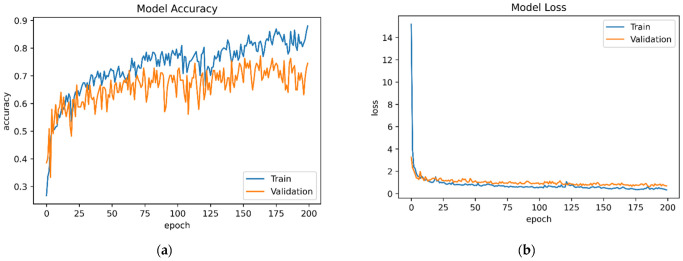
Deep Learning Evaluation Using DS1: (**a**) TModel1 Accuracy and (**b**) TModel1 Loss.

**Figure 23:**
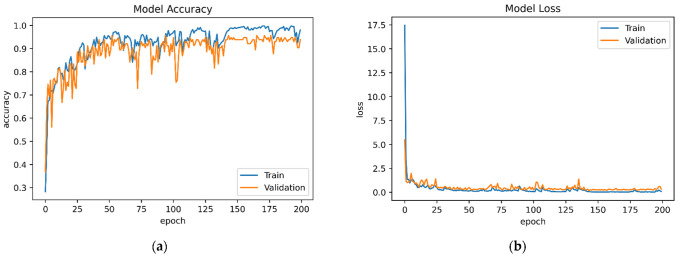
Deep Learning Evaluation Using DS2: (**a**) TModel2 Accuracy and (**b**) TModel2 Loss.

The TModel1 and TModel2 accuracy and loss results were plotted throughout each period and are presented in Figure 22 and Figure 23, respectively. Using different datasets, we can see the significant differences in performance. The TModel2 trained model, which is used for DS2, has a 98.01 percent training accuracy and a model loss of 0. 0883 percent. The model accuracy for the test dataset was 96.49 and the test model loss was 0.3672. The model accuracy of the TModel1 is 88.05 and it has a loss of 0.3374. The test model accuracy is 76.32 and it has a loss of 0.7190.

**Figure 24:**
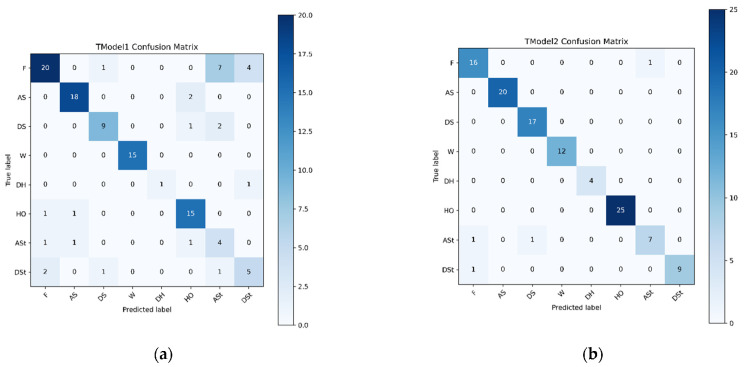
Deep Learning Confusion Matrix on the Test Dataset: (**a**) TModel1 and (**b**) TModel2.

Figure 24 plots the confusion matrices of the TModel1 and TModel2 deep learning models used on a test dataset that the trained model was not exposed to. For TModel1, the highest number of true positives were obtained for the Floor (20) and Ascending Stairs (18), followed by Wall and High Obstacle (15), Descending Stairs (9), Descending Step (5), Ascending Step (4), and Deep Hole (1). The total number of wrong predictions for the classes are Floor (13), Ascending Stairs (2), Descending Stairs (3), Deep Hole (1), High Obstacle (2), Ascending Step (3), and Descending Step (4). Wall was classified correctly. The Floor was misclassified 1 time as Descending Stairs, 7 times as Ascending Step, and 4 times as Descending Step. Ascending Step was misclassified 2 times as High Obstacle. Descending Stairs has been misclassified 1 time as High Obstacle and 2 times as Ascending Step. Deep Hole had one misclassification as Descending Step. High Obstacle was misclassified 1 time as Floor and 1 time as Ascending Stairs. Ascending Step was misclassified 1 time as Floor, 1 time as Ascending Stairs, and 1 time as High Obstacle. Descending Step was misclassified 2 times as Floor, 1 time as Descending Stairs, and 1 time as Ascending Step.

For TModel2, the highest number of true positives was obtained for the High Obstacle (25) and Ascending Stairs (20) classes, followed by Descending Stairs (17), Wall (12), Descending Step (9), Ascending Step (7), and Deep Hole (4). The total number of wrong predictions for the classes are Floor (1), Ascending Step (2), and Descending Step (1). Note that the Floor was misclassified 1 time as Ascending Stairs. Ascending Step was misclassified 1 time as Floor and 1 time as Descending Stairs. Descending Step was misclassified 1 time as Floor. Ascending Stairs, Descending Stairs, Wall, Deep Hole, and High Obstacle had no misclassifications.

## 6. Conclusions

In this paper, we developed the LidSonic V2.0 system by leveraging a comprehensive understanding of the state-of-the-art requirements and solutions involving assistive technologies for the visually impaired through a detailed literature review and a survey. We explained in Section 1 that it is difficult for visually impaired people to orient themselves and move in an unfamiliar environment without assistance and, hence, the focus regarding the LidSonic system in this paper is placed on the mobility tasks of the visually impaired. The system is based on a novel approach using a combination of a LiDAR with a servo motor and an ultrasonic sensor to collect data and predict objects using machine and deep learning for environment perception and navigation. The deep learning model TModel2, for the DS2 dataset, provided the overall best accuracy results at 96.49%. The second-best accuracy was provided by the KStar classifier at 95.44%, with a precision of 95.6%. The IBk and RC classifiers provided the same precision at 95.2% and similar accuracy results at 95.15% and 95%, respectively, using the DS2 dataset. Note that the IBk classifier was seen to be relatively non-dependent on the size of the datasets. This could be because both the datasets are numeric, and the difference between their sizes is small. It took 1 ms to train both the D2 and D1 datasets, and these were the fastest training times overall. The IBk classifier also provided the second fastest prediction time at 0.8 ms and, hence, we recommend using it at the edge for training and prediction. As for the KStar classifier, the training time is influenced by the size of the training dataset. It took 11 ms to train KStar with the DS2 dataset and 42 ms to train it with the larger DS1 dataset. Moreover, the KStar classifier required much longer times for the prediction, ranging between 22 ms and 55 ms, compared to the other classifiers in our experiments. Hence, we proposed using the KStar classifier at the fog or cloud layers.

We evaluated the proposed system from multiple perspectives. For instance, we proposed, based on the results, using the Random Committee classifier at the edge for prediction due to its faster prediction time, although it needs to be trained at the fog or cloud layers because it requires larger resources. In this respect, we plan to extend and integrate this work with other strands of our work on big data analytics and edge, fog, and cloud computing [148,149,150,151]. For example, we plan to experiment with different machine learning and deep learning methods at the edge, fog, and cloud layers, assessing their performance and the applicability of the use of edge, fog, and cloud computing for smart glasses, and considering new applications for the integration of smart glasses with cloud, fog, and edge layers. Another direction of our research is green and explainable AI [152,153], and we will also explore the expandability of the LidSonic system.

We created the second prototype of our LidSonic system in this work. The team constructed and tested the prototype. We also benefitted from the assistance of four other people aged 18 to 47 (who were not visually impaired) who helped to test and evaluate the LidSonic system. The time required to explain the device’s operation to the selected users was only a few minutes, but it varied depending on the user’s age and digital affinity. The tests were carried both indoors and outdoors on the campus of King Abdulaziz University. The purpose of this paper was to put the system’s machine learning and other technical capabilities to the test. Future work will involve testing the device with blind and visually impaired users so as to provide more details about the LidSonic system’s usability, human training, and testing aspects.

We conclude this paper with the remark that the technologies developed in this study show a high potential and are expected to open new directions for the design of smart glasses and other solutions for the visually impaired using open software tools and off-the-shelf hardware.

## Figures and Tables

**Table 2:** System Aspects and a Comparison with Related Works.

Research	Technology	Environment		Transparent Object Detection	Handsfree	Functioning in Dark	ML/DL	Vocal Feedback	High-Speed Processing	Low Energy Consumption	Low Cost	Low Memory Usage	Lightweight
		Indoor	Outdoor										
[133]	Solid-state LiDAR Sensor, RealSense L515 (LiDAR Depth Camera), Laptop	✓	✗	✗	✓	-	✓	✓	✗	✗	✗	✗	✓
[47]	LiDARs, Vibrotactile Units	✓	✓	✗	✓	✗	✓	✓	✗	✗	✓	✗	✓
[134]	Ultrasonic, PIR Motion Sensor, Accelerometer, Smartphone	✓	✓	✓	✓	✓	✗	✓	✓	✓	✓	✓	✓
This Work	TF-mini LiDAR, Ultrasonic	✓	✓	✓	✓	✓	✓	✓	✓	✓	✓	✓	✓

**Table 3:** Obstacle Dataset.

Image Class	No. of Instances	Example	Image Class	No. of Instances	Example
Floor	111	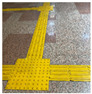	Deep Hole	20	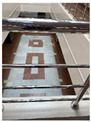
Ascending Stairs	108	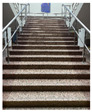	High Obstacle	149	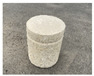
Descending Stairs	89	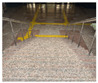	Ascending-Step/Bump	62	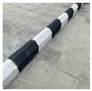
Wall	87	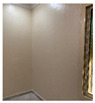	Descending-Step/Hole	34	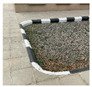

**Table 4:** Preprocessing and Feature Extraction.

Name	Preprocessing and Feature Extraction
PF1	Adjust the upwards lines of the readings to be the same (angle index) with the downwards readings
PF2	PF1 Extract 11 angle reading by dividing the 60 readings by 10 + angle no. 60 Add three features: Calculate the height of the obstacle of the starting angle of the LidSonic V2.0 device (angle closest to the user h1) Calculate the height of the obstacle of the middle angle of the LidSonic V2.0 device scan (h2) Calculate the slope between h1 and h2

**Table 5:** Dataset 1 (D1) Sample.

Obstacle Type	Data
DS1 Labels	a01, a02, a03, a04, a05, a06, a07, a08, a09, a10, a11, a12, a13, a14, a15, a16, a17, a18, a19, a20, a21, a22, a23, a24, a25, a26, a27, a28, a29, a30, a31, a32, a33, a34, a35, a36, a37, a38, a39, a40, a41, a42, a43, a44, a45, a46, a47, a48, a49, a50, a51, a52, a53, a54, a55, a56, a57, a58, a59, a60, Obstacle_class
Floor	309, 305, 301, 295, 288, 285, 274, 266, 264, 260, 259, 253, 249, 243, 240, 229, 227, 211, 215, 214, 211, 208, 208, 205, 205, 197, 193, 191, 187, 186, 180, 176, 172, 169, 167, 172, 173, 172, 171, 169, 168, 167, 166, 165, 164, 163, 161, 161, 159, 158, 33, 25, 24, 23, 23, 26, 29, 120, 154, 157, 0
Ascending Stairs	141, 143, 143, 142, 143, 127, 145, 145, 142, 143, 144, 146, 147, 145, 141, 129, 133, 134, 127, 130, 134, 135, 135, 134, 134, 134, 134, 133, 131, 130, 128, 127, 124, 123, 122, 121, 119, 118, 119, 121, 130, 130, 129, 129, 128, 128, 124, 123, 124, 123, 123, 123, 122, 122, 132, 133, 132, 131, 133, 133, 1
Descending Stairs	696, 740, 841, 834, 575, 372, 380, 396, 608, 659, 654, 614, 546, 353, 343, 386, 389, 385, 382, 374, 359, 356, 355, 354, 350, 342, 336, 321, 308, 305, 303, 299, 296, 268, 264, 264, 262, 264, 263, 261, 254, 190, 189, 201, 215, 217, 211, 211, 209, 207, 208, 208, 206, 200, 154, 186, 186, 186, 185, 183, 2
Wall	49, 52, 46, 46, 46, 50, 51, 50, 52, 49, 53, 52, 50, 50, 55, 54, 57, 55, 55, 57, 58, 56, 57, 58, 62, 62, 62, 63, 65, 64, 66, 66, 69, 70, 78, 80, 83, 83, 84, 86, 88, 93, 95, 96, 102, 111, 123, 126, 118, 120, 120, 120, 122, 125, 129, 141, 152, 152, 152, 152, 3
Deep Hole	638, 643, 646, 650, 654, 659, 654, 661, 669, 663, 666, 668, 669, 672, 638, 631, 628, 630, 592, 588, 589, 592, 577, 554, 555, 532, 531, 531, 530, 528, 523, 520, 495, 494, 490, 487, 485, 482, 480, 476, 472, 450, 441, 443, 446, 438, 434, 434, 434, 436, 436, 430, 429, 427, 426, 423, 422, 422, 422, 421, 4
High Obstacle	87, 85, 85, 85, 85, 84, 84, 85, 89, 88, 91, 93, 93, 94, 99, 99, 100, 100, 98, 98, 98, 100, 100, 102, 103, 103, 103, 103, 102, 101, 99, 98, 96, 96, 95, 94, 93, 92, 91, 91, 91, 90, 88, 85, 86, 86, 84, 80, 78, 77, 77, 81, 81, 81, 81, 81, 81, 79, 78, 75, 5
Bump	237, 225, 217, 231, 239, 242, 227, 219, 219, 220, 228, 205, 205, 207, 210, 212, 212, 200, 196, 196, 196, 195, 194, 183, 184, 176, 174, 173, 170, 168, 167, 166, 164, 163, 160, 159, 158, 157, 153, 151, 151, 147, 144, 144, 144, 148, 148, 148, 147, 144, 143, 143, 143, 143, 143, 143, 142, 141, 140, 139, 6
Hole	275, 275, 298, 298, 273, 276, 284, 294, 296, 263, 254, 260, 262, 262, 254, 252, 252, 251, 249, 248, 245, 243, 231, 227, 228, 215, 214, 212, 212, 210, 209, 206, 203, 201, 201, 201, 200, 196, 189, 191, 191, 186, 184, 184, 185, 180, 181, 177, 178, 177, 178, 175, 173, 174, 174, 175, 174, 172, 171, 171, 7

**Table 6:** Dataset 2 (D2) Sample.

Obstacle Type	Data
DS2 Labels	a01, a06, a12, a18, a24, a30, a36, a42, a48, a54, a60, h1, h2, s, Obstacle_class
Floor	309, 285, 253, 211, 205, 186, 172, 167, 161, 23, 157, 0.96, 2.42, 0.02, 0
Ascending Stairs	141, 127, 146, 134, 134, 130, 121, 130, 123, 122, 133, 24.21, 47.76, 0.54, 1
Descending Stairs	696, 372, 614, 385, 354, 305, 264, 190, 211, 200, 183, −24.20, −93.90, −0.55, 2
Wall	49, 50, 52, 55, 58, 64, 80, 93, 126, 125, 152, 5.81, 101.19, 19.06, 3
Deep Hole	638, 659, 668, 630, 554, 528, 487, 450, 434, 427, 421, −254.67, −274.42, −0.09, 4
High Obstacle	87, 84, 93, 100, 102, 101, 94, 90, 80, 81, 75, 80.37, 71.23, −0.23, 5
Bump	236, 264, 221, 209, 182, 173, 162, 152, 148, 141, 139, 18.39, 12.95, −0.08, 6
Hole	275, 276, 260, 251, 227, 210, 201, 186, 177, 174, 171, −12.58, −17.0, −0.05, 7

**Table 7:** TensorFlow Training Model Shapes.

Dataset	TModel1	TModel2
Training feature shape	(435, 60)	(435, 14)
Validation feature shape	(109, 60)	(109, 14)
Test feature shape	(136, 60)	(136, 14)

**Table 8:** TModel1 Summary.

Model: “Sequential_16”		
Layer (Type)	Output Shape	Parameters
flatten_16 (Flatten)	(None, 60)	0
dense_74 (Dense)	(None, 60)	3660
dense_75 (Dense)	(None, 120)	7320
dense_76 (Dense)	(None, 60)	7260
dense_77 (Dense)	(None, 60)	3660
dense_78 (Dense)	(None, 30)	1830
dense_79 (Dense)	(None, 8)	248
Total params: 23,978		
Trainable params: 23,978		
Non-trainable params: 0		

**Table 9:** TModel2 Summary.

Model: “Sequential_2”		
Layer (Type)	Output Shape	Parameters
flatten_2 (Flatten)	(None, 14)	0
dense_8 (Dense)	(None, 140)	2100
dense_9 (Dense)	(None, 64)	9024
dense_10 (Dense)	(None, 30)	1950
dense_11 (Dense)	(None, 8)	248
Total params: 13,322		
Trainable params: 13,322		
Non-trainable params: 0		

**Table 10:** Deep Learning Model Configurations.

Specifications	Value
Number of hidden layers—Tmodel1	5
Number of hidden layers—Tmodel2	3
Activation function—hidden layers	Relu
Activation function—output layer	Softmax
Loss function categorical	Sparse_categorical_crossentropy
Optimizer	Adam
Epochs	200

**Table 11:** Evaluation of the Machine Learning Models.

Trained Model	Dataset	No. of Features	Classifier	Precision	Accuracy
IBk-DS1	DS1	60	IBk	94.0	93.68
RC-DS1	DS1	60	Random Committee	94.7	94.56
KStar-DS1	DS1	60	KStar	95.6	95.44
IBk-DS2	DS2	14	IBk	95.2	95
RC-DS2	DS2	14	Random Committee	95.2	95.15
KStar-DS2	DS2	14	KStar	94.1	93.82

**Table 12:** Class Abbreviation.

Class	Abbreviation	Class	Abbreviation
Floor	F	Deep Hole	DH
Ascending Stairs	AS	High Obstacle	HO
Descending Stairs	DS	Ascending Step	Ast
Wall	W	Descending Step	DSt

**Table 13:** TensorFlow Model Evaluation.

Trained Model	Dataset	Features	Model Accuracy	Test Accuracy	Model Loss	Test Loss
TModel1	D1	60	88.05	76.32	0.3374	0.7190
TModel2	D2	14	98.01	96.49	0.0883	0.3672

## Data Availability

The dataset developed in this work can be provided on request.

## Abbreviations

The following abbreviations are used in this paper.

ETA	Electronic Travel Aids
ToF	Time-of-flight
RNA	Robotic Navigation Aid
ALVU	Array of LiDARs and Vibrotactile Units
BLE	Bluetooth Low Energy
SDK	Software Development Kit
RGB-D	RGB-Depth
CNN	Convolutional Neural Networks
RFID	Radio Frequency Identification Reader
HRTFs	Head-Related Transfer Functions
GPS	Global Positioning System
MSER	Maximally Stable External Region
SWT	Stroke Width Transform
